# Piezoelectric dual-network tough hydrogel with on-demand thermal contraction and sonopiezoelectric effect for promoting infected-joint-skin-wound healing via FAK and AKT signaling pathways

**DOI:** 10.1093/nsr/nwaf118

**Published:** 2025-03-29

**Authors:** Jinlong Luo, Zhen Liang, Xin Zhao, Shengfei Huang, Yanan Gu, Zexing Deng, Jing Ye, Xingmei Cai, Yong Han, Baolin Guo

**Affiliations:** State Key Laboratory for Mechanical Behavior of Materials, and Frontier Institute of Science and Technology, Xi'an Jiaotong University, Xi'an 710049, China; Department of Plastic Surgery, Xijing Hospital, Fourth Military Medical University, Xi'an 710032, China; State Key Laboratory for Mechanical Behavior of Materials, and Frontier Institute of Science and Technology, Xi'an Jiaotong University, Xi'an 710049, China; State Key Laboratory for Mechanical Behavior of Materials, and Frontier Institute of Science and Technology, Xi'an Jiaotong University, Xi'an 710049, China; Department of Plastic Surgery, Xijing Hospital, Fourth Military Medical University, Xi'an 710032, China; College of Materials Science and Engineering, Xi'an University of Science and Technology, Xi'an 710054, China; State Key Laboratory for Mechanical Behavior of Materials, and Frontier Institute of Science and Technology, Xi'an Jiaotong University, Xi'an 710049, China; State Key Laboratory for Mechanical Behavior of Materials, and Frontier Institute of Science and Technology, Xi'an Jiaotong University, Xi'an 710049, China; State Key Laboratory for Mechanical Behavior of Materials, and Frontier Institute of Science and Technology, Xi'an Jiaotong University, Xi'an 710049, China; Department of Orthopaedics, The First Affiliated Hospital of Xi'an Jiaotong University, Xi'an 710061, China; State Key Laboratory for Mechanical Behavior of Materials, and Frontier Institute of Science and Technology, Xi'an Jiaotong University, Xi'an 710049, China; Department of Orthopaedics, The First Affiliated Hospital of Xi'an Jiaotong University, Xi'an 710061, China; Department of Dermatology, The Second Affiliated Hospital of Xi'an Jiaotong University, Xi'an 710004, China

**Keywords:** on-demand thermal contraction, piezoelectricity, sonopiezoelectric therapy, dynamic wound management, chronic joint-skin wound

## Abstract

The dynamic and whole stage management of infected wound healing throughout the entire repair process, including intelligent on-demand wound closure and the regulation of the transition from bactericidal to reparative phases, remains a major challenge. This study develops sonopiezoelectric-effect-mediated on-demand reactive-oxygen-species release by incorporating piezoelectric barium titanate modified with gold nanoparticles and a thermally responsive dual-network tough hydrogel dressing with a physical network structure based on ureidopyrimidinone-modified gelatin crosslinked by multiple hydrogen bonds, and with a chemical network structure based on N-isopropylacrylamide and methacryloyl gelatin formed via radical polymerization. This hydrogel exhibits temperature-sensitive softening, on-demand thermal contraction performance, high mechanical strength, good tissue adhesion, outstanding piezoelectricity, tunable sonopiezoelectric behavior, regulatable photothermal properties and desirable biocompatibility. The tunable sonopiezoelectric effect enables the hydrogel to eliminate wound bacteria in the short term, and effectively promote human fibroblast proliferation and migration over the long term. The hydrogel dressing actively contracts to close wound edges and further promotes the healing of MRSA-infected skin defects in the neck of mice by promoting fibroblast migration, enhancing collagen deposition and facilitating angiogenesis via up-regulating the FAK and AKT signaling pathways, providing a novel design strategy for developing dressings targeting chronic joint-skin wounds.

## Teaser

A sonopiezoelectric ROS-releasing hydrogel integrates on-demand thermal contraction, local electrical stimulation, and whole-process management to dynamically switch bactericidal-reparative phases, accelerating MRSA-infected chronic wound healing.

## INTRODUCTION

The skin, the largest organ of the human body, serves as the first line of defense and plays a crucial role in maintaining homeostasis and supporting normal physiological activities [1]. Consequently, the skin is frequently exposed to damage from physical, chemical or biological factors, making it one of the most vulnerable organs [2–4]. Upon injury, the skin orchestrates the activation and guidance of various cells and bioactive molecules to repair the damage, with the goal of tissue regeneration and functional remodeling of the affected area [5–7]. However, wound infection can lead to persistent inflammation, impaired angiogenesis, excessive exudate and accumulation of reactive oxygen species (ROS), resulting in chronic wounds and delayed wound healing, particularly for wounds in dynamic environments induced by variable mechanical stress [8–10]. Chronic wounds seriously affect the quality of life of patients [11–13]. Due to their extracellular matrix-mimicking structure, combination of multiple functions, and convenient carrying of drug/cell/bioactive molecules, hydrogels are considered ideal candidates for wound dressings [14,15]. Multifunctional hydrogels have been continuously developed to facilitate wound repair, and conductive hydrogels are regarded as one of the most representative types. Researchers worked to optimize the conductive hydrogel's conductivity, sensitivity, mechanical stability, durability and biocompatibility to make it more effective in promoting skin healing. However, the mode of action of conductive hydrogels is usually passive, such as transmitting biological signals, and the lack of active stimulation function has met the demand for on-demand treatment under complex wound conditions [16]. Natural polymeric hydrogels such as hyaluronic acid-based hydrogels are often considered for use in dressings to promote skin-wound repair, due to their excellent biocompatibility and inherent biological activity [17]. However, their unstable mechanical properties and single cross-linked network seriously restrict their further development [18]. Existing hydrogels also face the challenge of integrating intelligent wound-repair management. This involves the need to combine on-demand wound closure in the initial stage with the transition from antibacterial and anti-inflammatory actions to promoting wound repair in subsequent stages, all within a single hydrogel platform.

Dynamic chronic wounds, under various mechanical deformations, struggle to heal, primarily due to difficulties in wound closure, which leads to a vicious cycle of persistent infection and inflammation. Recent research indicates that dressings with active contraction can draw wound edges together and significantly promote fibroblast proliferation and migration through mechanical stress, thereby accelerating chronic diabetic wound healing [19]. Currently, contracting hydrogel dressings are categorized as water-triggered, acid-triggered and/or light-triggered shape memory contracting dressings [19,20], and thermoresponsive phase-transition-triggered contracting dressings [21–23]. However, these shape-memory-induced contraction dressings are limited by their ‘disposable’ nature, making them unsuitable for on-demand wound closure management [24,25]. On the other hand, although the thermo-induced phase-transition contracting dressings could display limited on-demand contraction, the main issues with thermo-induced phase-transition contracting dressings are: (i) thermoresponsive contraction increases hydrogel strength, hindering further contraction. More importantly, the hydrogel wound returns to its original shape after temperature cooling, limiting contraction effects. (ii) The inability to further adjust the post-contraction shape. These limitations greatly hinder their ability to manage wound closure on demand. Therefore, it is important to ameliorate the on-demand contraction behavior of thermo-induced phase-transition contracting dressings. Moreover, the dynamic chronic wound environment requires thermoresponsive contracting hydrogel dressings to possess sufficient toughness, enabling them to withstand various mechanical deformations without failure. The toughness of hydrogels can be enhanced through strategies such as employing multiple networks [26,27], utilizing macromolecular crosslinkers [28] and introducing sacrificial bonds [29]. However, to the best of our knowledge, no hydrogel wound dressing currently offers on-demand, long-term contraction combined with tough mechanical properties for use in dynamic chronic wounds.

Inflammation is the second stage of wound healing. Wound infection can lead to prolonged inflammation, ROS accumulation, delayed healing and potential death. Recent studies suggest that piezoelectric materials can efficiently kill bacteria by generating free electrons in response to mechanical stress, which subsequently react with water and oxygen to produce ROS [30]. As a special biological functional molecule, high levels of ROS recruit immune cells to eliminate bacteria at chronic wound sites, while physiological levels of ROS promote the proliferation, migration and differentiation of cells involved in wound repair, stimulate vasoconstriction, and induce platelet aggregation for hemostasis [31]. Studies indicate that ultrasound can induce mechanical deformation in piezoelectric materials such as barium titanate (BTO), leading to the generation of varying amounts of ROS depending on ultrasound intensity. Thus, incorporating piezoelectric materials into hydrogels enables on-demand ROS generation under external ultrasound stimulation. Furthermore, gold nanoparticles with excellent electrical conductivity and photothermal effects have been modified on the surface of BTO (BTO@Au) to form metal/semiconductor Schottky junctions, thereby enhancing BTO's piezoelectric catalytic performance [32]. Notably, generating high levels of ROS in the early stages of a dynamically infected wound can help kill bacteria and prevent inflammation, while low ROS levels in later stages benefit wound repair, thereby effectively promoting chronic wound healing. In addition, piezoelectric materials can not only release ROS controllably, but also generate a local micro electric field under external mechanical stress. This micro electric field can simulate the endogenous electric field generated after wound epithelial injury [33]. It can stimulate the proliferation and migration of human cells, and ultimately promote chronic wound repair by promoting granulation tissue formation and re-epithelialization [34]. Thus, the combination of hydrogel matrix with intelligent on-demand wound closure and tough mechanical properties, and piezoelectric and photothermal BTO@Au with intelligent regulation of transformation from bactericidal to repair within a single dressing platform, shows huge promise for the whole stage management of dynamic infected chronic wound repair, which is still a great challenge.

To achieve intelligent wound management, this study firstly designs and fabricates a dual-network hydrogel dressing (UNMx) based on thermoresponsive gelatin derivatives and poly(N-isopropylacrylamide) (PNIPAM). The first network comprises ureidopyrimidinone (UPy)-modified gelatin (GU), crosslinked via multiple hydrogen bonds between UPy groups, while the second network consists of N-isopropylacrylamide (NIPAM) and methacryloyl gelatin (GMA) polymerized under ammonium persulfate (APS) initiation. Then, after incorporating the piezoelectric material BTO@Au into the UNMx hydrogel matrix, the final hydrogel dressing UNMx/BAy (BA means BTO@Au) features integrated intelligent active thermoresponsive contraction and piezoelectric-mediated on-demand ROS release and local electrical stimulation functions to promote dynamic chronic wound healing. The ingenious heating (45°C) triggered by near-infrared light enhances the contraction ratio of the hydrogel, and subsequent cooling at room temperature fixes the contraction due to multiple hydrogen bonds, further improving the hydrogel's thermoresponsive contraction efficiency. With regulatable piezoelectric properties and stable thermal contraction, even after the temperature returns to a relatively low level, the hydrogel releases high levels of ROS for antibacterial effects under high ultrasound power and low levels of ROS and electrical stimulation to promote cell migration and angiogenesis under low ultrasound power, further facilitating chronic wound repair. Wound healing rates, collagen deposition measurements, histological examinations, laser Doppler imaging and immunofluorescence staining were conducted on MRSA (methicillin-resistant *Staphylococcus aureus*)-infected full-thickness joint-skin-defect models in mice to assess the hydrogel's efficacy in promoting dynamic chronic wound healing.

## RESULTS AND DISCUSSION

### Synthesis and characterization of hydrogel

**Figure 1.**
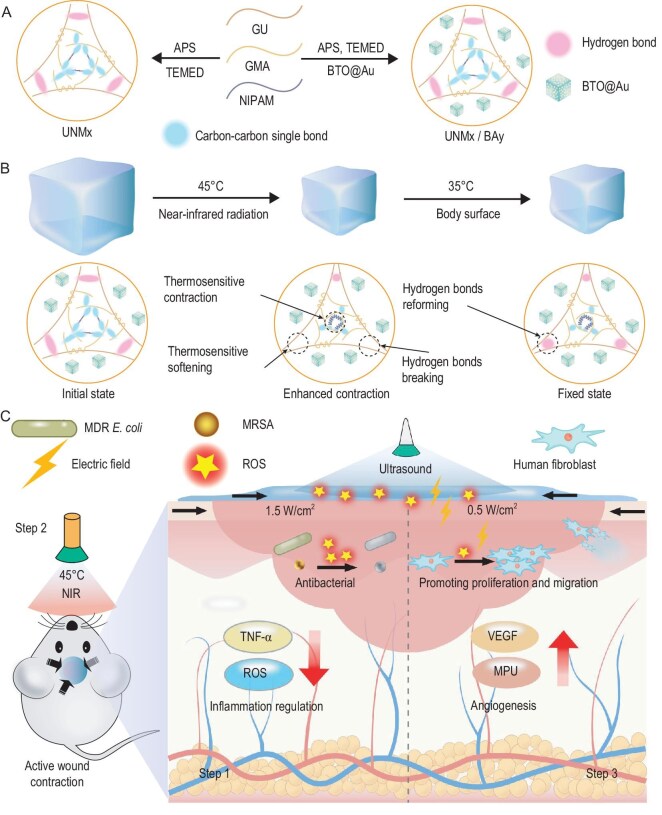
Schematic of hydrogel preparation, mechanism of enhanced thermoresponsive contraction and application in chronic infected wounds. (A) The preparation process of UNMx and UNMx/BAy hydrogels, where U represents GU, N represents NIAPM, M represents GMA, BA represents BTO@Au, x represents the concentration of GMA and y represents the concentration of BTO@Au, both expressed in mg/mL. (B) The principle of enhancing hydrogel thermoresponsive contraction performance by increasing contraction efficiency upon heating and fixing contraction upon cooling. (C) A schematic of the mechanism by which the hydrogel treats chronic infected wounds.

This study designs and fabricates a double-network hydrogel dressing with active thermoresponsive contraction and piezoelectric properties based on gelatin derivatives and PNIPAM to promote the healing of chronically infected wounds. Gelatin, one of the most widely used natural polymers, exhibits excellent biocompatibility. The backbone of gelatin contains RGD (Arg-Gly-Asp) sequences that promote cell adhesion, thereby favoring cell proliferation and migration, making it an ideal raw material for designing bioactive materials [35,36]. Additionally, gelatin can be modified or processed to produce tough hydrogels, which is typically achieved through chemical crosslinking (such as glutaraldehyde), blending with other polymers (polyvinyl alcohol) or incorporating functional groups (methacrylation) [37]. Given these properties, gelatin was chosen as the base material for hydrogel dressings. As shown in Fig. 1A, in constructing the double network of the hydrogel, UPy-modified GU forms the first network through multiple hydrogen bonds between UPy groups, while NIPAM and GMA are polymerized through free radical polymerization initiated by APS to form the second network. Additionally, BTO nanocubes loaded with gold nanoparticles (BTO@Au) were incorporated into the hydrogel matrix to provide photothermal, piezoelectric effects and improved conductivity.

The hydrogels prepared in this study exhibit active thermoresponsive contraction, which, under photothermal action, exerts contractile force to pull the wound edges toward closure, thereby accelerating wound healing. Figure 1B illustrates the principle of thermoresponsive contraction of the UNMx/BAy hydrogel. The contraction force of the hydrogel network originates from the PNIPAM polymer chains. GMA, as a macromolecular crosslinker, co-polymerizes with NIPAM to crosslink the second network while entangling with and forming hydrogen bonds to the first network via macromolecular chains, further enhancing the hydrogel's mechanical robustness. In this design, the dual-network hydrogel contracts when subjected to external heat stimulation due to the contraction of PNIPAM chains, pulling the entire network into collapse and resulting in the hydrogel's thermoresponsive contraction. Typically, PNIPAM-based hydrogels that contract under heat stimuli gradually recover their volume upon cooling, failing to maintain the contraction ratio and effect. To overcome this limitation, we designed a mechanism that enhances the contraction ratio after heating, due to the breaking of hydrogen bonds, while maintaining and fixing the hydrogel's contraction through subsequent cooling. Specifically, under near-infrared light, the hydrogel's temperature is controlled at 45°C, causing multiple hydrogen bonds in GU to break, softening the hydrogel and enhancing contraction ratio and efficiency. Upon cessation of near-infrared irradiation, the hydrogel cools down to body surface temperature (∼35–37°C), reforming multiple hydrogen bonds, thereby fixing the thermoresponsive contraction.

The incorporation of BTO@Au endows the hydrogel with photothermal, piezoelectric effects and improved conductivity. Relevant studies have shown that metal/semiconductor Schottky junctions can be formed when gold nanoparticles are reduced and modified on the surface of BTO nanocubes, which can promote electron-hole-pair separation and migration by inducing BTO band bending, thus improving the piezoelectric catalytic performance of BTO [32]. Due to the localized surface plasmon resonance of gold nanoparticles, BTO@Au provides excellent near-infrared absorption and photothermal effects [38]. Under near-infrared irradiation, the hydrogel dressing's temperature gradually rises due to the photothermal effect. By controlling the power density of the near-infrared laser, the hydrogel's temperature can be maintained at ∼45°C, ensuring the conditions required for thermoresponsive contraction. Studies have shown that a lower photothermal temperature of 45°C does not cause irreversible thermal damage to skin tissues, thereby avoiding additional injury during treatment [39]. The Schottky junction formed between gold nanoparticles and the piezoelectric semiconductor BTO endows the UNMx/BAy hydrogel with excellent piezoelectric properties [32]. This piezoelectric effect can be triggered by external ultrasound stimulation, generating electrons that react with environmental oxygen and water molecules to form ROS, thereby making the UNMx/BAy hydrogel a superior sonopiezoelectric therapy material. As illustrated in Fig. 1C, this study employs a high ultrasound intensity (1.5 W/cm²) to generate high levels of ROS, effectively addressing drug-resistant bacterial infections in skin wounds at the early stage. In the second step, under near-infrared irradiation, the hydrogel dressing actively contracts, pulling the wound edges closer to reduce the wound area. Finally, to enhance the hydrogel's effectiveness in promoting the healing of chronic motion-related wounds, a low-intensity ultrasound (0.5 W/cm²) is applied, generating a localized micro electric field and low levels of ROS. Among them the gold nanoparticles on the surface of BTO significantly increase the hydrogel's conductivity, facilitating electrical stimulation. Even in the absence of ultrasound, the deformation of the hydrogel itself can induce micro electric field stimulation from the piezoelectric material, further aiding wound repair. The localized micro electric field and low levels of ROS help to decrease inflammation, and promote the proliferation, migration and angiogenesis of functional cells, thereby supporting dynamic wound healing [40].

The chemical structures of the polymers and hydrogels were initially characterized. UPy was synthesized through the reaction between the amino group of 2-amino-4-hydroxy-6-methylpyrimidine (AHMT) and the isocyanate group of hexamethylene diisocyanate (HDI) (Fig. S1A). Its chemical structure was confirmed by ¹H nuclear magnetic resonance (NMR) spectroscopy, with hydrogen atoms in various chemical environments labeled (Fig. S2A). GU was synthesized using UPy to modify gelatin (Fig. S1B). Figure S2B displays the ¹H NMR spectrum of GU, where the characteristic peaks at 2.1 ppm and 5.7 ppm correspond to the methyl group on UPy and the hydrogen atoms on the pyrimidine ring, respectively, indicating successful grafting of UPy onto gelatin and thus confirming the preparation of GU. GMA was synthesized by modifying gelatin with methacrylate anhydride (Fig. S1C). In Fig. S2C, the characteristic peaks representing methacryloyl groups at 5.3 ppm and 5.7 ppm further confirm the preparation of GMA. Fourier transform infrared (FT-IR) spectroscopy was employed to further investigate the chemical structures of the polymers and hydrogels. As shown in Fig. S2I, compared with gelatin (GT), the FT-IR spectra of GU, GMA and UNM10 hydrogel exhibit three specific characteristic peaks. The peak near 2850 cm⁻¹ is attributed to the symmetric stretching vibration of C-H in the methyl groups of UPy and methacryloyl groups; the peak near 1380 cm⁻¹ is due to the bending vibration of C-H in these methyl groups, and the peak near 1160 cm⁻¹ is attributed to the stretching vibration of C-N in ureido or amide bonds.

The microstructure of BTO@Au was observed using high-resolution transmission electron microscopy (HRTEM). As shown in Fig. S2D, BTO appears as nanocubes with gold nanoparticles uniformly dispersed on its surface, and the particle size of BTO@Au is ∼100 nm. In the HRTEM image (Fig. S2E), the lattice spacing of 0.405 nm corresponds to the (001) plane of BTO, while the lattice spacing of 0.235 nm corresponds to the (111) plane of gold nanoparticles. In the selected area electron diffraction image (Fig. S2F), diffraction rings corresponding to the lattices of BTO and gold nanoparticles appear, consistent with the perovskite structure of BTO and the face-centered cubic structure of gold. Subsequently, elemental mapping of BTO@Au was analyzed using energy-dispersive X-ray spectroscopy (EDS). Both line scan (Fig. S2G and J) and area scan (Fig. S2H) confirmed the presence of oxygen, titanium, barium and gold. Finally, X-ray diffraction (XRD) was used to characterize BTO@Au, further confirming the perovskite structure of BTO and the face-centered cubic structure of gold (Fig. S2K).

### Rheological properties, tensile strength and adhesive strength of UNMx hydrogel

**Figure 2.**
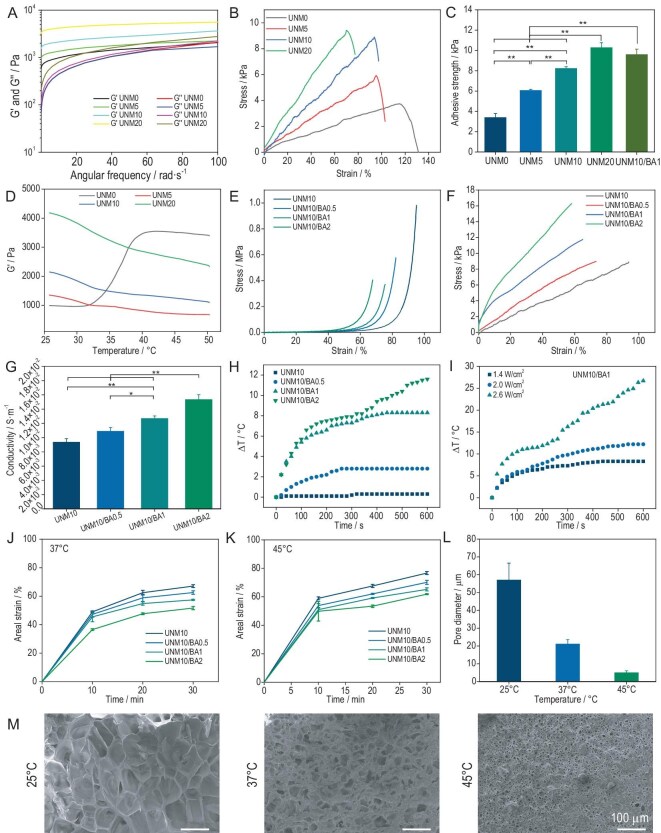
Characterization and performance of UNMx/BAy hydrogel. (A) Rheological properties of UNMx hydrogel under varying frequencies. (B) Tensile stress-strain curves of UNMx hydrogel. (C) The tissue adhesion strength of UNMx/BAy hydrogel on pigskin. (D) Rheological properties of UNMx hydrogel under varying temperatures. (E) Uniaxial compressive stress-strain curves of UNMx/BAy hydrogel. (F) Tensile stress-strain curves of UNMx/BAy hydrogel. (G) Electrical conductivity of UNMx/BAy hydrogel. (H) ΔT-time curves under fixed power (1.4 W/cm²) near-infrared laser treatment. (I) ΔT-time curves under different-power near-infrared laser treatments for UNM10/BA1. (J) Surface strain of hydrogel under thermal contraction at 37°C. (K) Surface strain of hydrogel under thermal contraction at 45°C. (L) The pore diameter of UNM10/BA1 at different temperatures. (M) Representative SEM images of UNM10/BA1 at different temperatures. *P < 0.05, **P < 0.01.

Wound dressings must exhibit sufficient mechanical strength to seal wounds and maintain environmental stability. GMA was employed as a macromolecular crosslinker for the UNMx hydrogel dressing, forming a polymer network through chemical crosslinking with PNIPAM, combined with molecular entanglement and hydrogen bonding with GU. In this study, the GMA content was varied to investigate its impact on the rheological properties of the hydrogel dressing. As shown in Fig. 2A, the effect of GMA concentration on the rheological properties was evaluated by measuring the storage modulus of the hydrogel dressing. At an angular frequency of 10 rad/s, the storage modulus of UNM0, which did not contain GMA, was the lowest at ∼983 Pa. As GMA content increased from 5 mg/mL to 10 mg/mL and 20 mg/mL, the storage moduli of UNM5, UNM10 and UNM20 increased from 1354 Pa to 2124 Pa and 4179 Pa, respectively. Additionally, across the angular frequency range of 10 rad/s to 100 rad/s, the storage modulus of the hydrogel dressing remained higher than its loss modulus, indicating that the hydrogel dressing could withstand shear damage to a certain extent while maintaining its structural integrity. Rheological tests confirmed that the introduction of GMA as a macromolecular crosslinker significantly enhanced the strength of the hydrogel, with greater enhancement observed at higher GMA content.

The implementation of active thermoresponsive contraction in hydrogel dressings to accelerate wound closure requires the hydrogel to exhibit excellent toughness. Good toughness enhances material compliance, leading to better wound conformity. Tensile tests were conducted to evaluate the impact of GMA content on the toughness of UNMx hydrogel dressings (Fig. 2B). The corresponding value of toughness for UNM0, UNM5, UNM10 and UNM20 was 2.33 kJ/M^3^, 2.85 kJ/M^3^, 4.16 kJ/M^3^ and 3.51 kJ/M^3^, respectively. Among them, the UNM10 sample has the highest toughness because it has comparable highest tensile strength while maintaining desirable tensile strain. UNM0, which did not contain GMA, exhibited the highest fracture strain at 116%, corresponding to the lowest fracture stress of 3.71 kPa. As the GMA content increased, the fracture strain of the UNMx hydrogel dressings decreased, while the fracture stress increased, attributed to the increased crosslinking density of the hydrogel network at higher GMA content. Notably, UNM10 displayed only a 2% reduction in fracture strain compared to UNM5, while its fracture stress increased by nearly 2 kPa. In summary, the UNMx hydrogel dressings exhibited excellent toughness due to the unique network structure design. The double-network design is among the most common strategies to enhance hydrogel toughness [41–43]. The multiple hydrogen bonds in GU provide a sacrificial bond strategy for the first network, consuming energy from external stress stimuli and enhancing hydrogel strength. Additionally, GMA, as a macromolecular crosslinker, introduces more mobile chain segments into the hydrogel network, further enhancing its toughness [44]. Moreover, the rich molecular chain entanglements and hydrogen bonds between GMA and GU further contribute to the improved toughness of the hydrogel [45].

The functionality of active thermoresponsive contraction to promote wound closure requires the hydrogel to exhibit adequate tissue adhesive strength. Lap shear tests were conducted to measure the shear adhesive strength of UNMx hydrogel dressings on pigskin (Fig. 2C, Figs S3 and S4). The tissue adhesion strength of UNM10/BA1 reached 9.59 kPa, which was significantly higher than the 8.22 kPa of UNM10 (Fig. 2C). Compared with UNM10, the interface adhesion mechanism of UNM10/BA1 has no change, and the improvement of its adhesion strength is attributed to the higher material cohesiveness strength endowed by BTO@Au, which has been verified by uniaxial compression and tensile experiments. As the GMA content increased from 0 to 5 mg/mL, 10 mg/mL and 20 mg/mL, the tissue adhesive strength of UNM0, UNM5, UNM10 and UNM20 increased from 3.39 kPa to 6.06 kPa, 8.22 kPa and 10.27 kPa, respectively. The tissue adhesive strength of hydrogel dressings is determined by both interface adhesive strength and cohesion. The interface adhesive strength of UNMx hydrogel dressings is primarily provided by the rich hydrogen bonds and minor carbon–carbon double bonds forming chemical crosslinks with the amino and thiol groups at the tissue interface, resulting in no significant differences [46]. The increased GMA content enhances the toughness of UNMx hydrogel dressings, providing better cohesion and improved tissue adhesive strength.

To further optimize the active thermoresponsive contraction of hydrogel dressings for wound closure, a mechanism was designed using multiple hydrogen bonds in GU to enhance contraction efficiency upon heating and fix contraction upon cooling. As shown in Fig. 2D, rheological tests under variable temperature conditions were conducted to validate this design. Overall, at room temperature (25°C), the storage moduli of hydrogel dressings increased with higher GMA content. UNM5, UNM10 and UNM20, which contained GMA, exhibited a decrease in storage modulus as the temperature increased. The decrease in modulus with increasing temperature is attributed to the breaking of multiple hydrogen bonds in GU, enhancing the contraction efficiency of the hydrogel dressings. When the temperature returned to mouse skin temperature (∼35°C), the PNIPAM molecular chains remained collapsed, as the temperature was still above their lower critical solution temperature (32°C). The reformation of hydrogen bonds in GU increased the modulus, fixing the contracted state of the hydrogel and preventing the contraction ratio from decreasing, thereby improving thermoresponsive contraction efficiency. Notably, UNM0, which did not contain GMA, exhibited a sharp increase in storage modulus at 32°C, followed by a slow decrease after 40°C. This is because, below 40°C, changes in hydrogel modulus are primarily dominated by the collapse of PNIPAM, which pins the polymer chains and limits their movement, thereby increasing storage modulus. For UNM5, UNM10 and UNM20 containing GMA, the collapse of PNIPAM was influenced by GMA crosslinking, requiring the contraction of the entire hydrogel network, thereby limiting contraction and not significantly affecting the storage moduli. Above 40°C, the increased breaking of multiple hydrogen bonds in GU became the dominant factor affecting the storage moduli of hydrogel dressings, resulting in a slow decrease in storage modulus with increasing temperature.

Based on a comprehensive study of the rheological properties, tensile strength and tissue adhesive strength of UNMx hydrogel dressings, UNM10 was selected for subsequent studies due to its optimal overall performance.

### Mechanical, conductive and biocompatible properties of UNMx/BAy hydrogel

The addition of BTO@Au during the preparation process results in the formation of UNMx/BAy hydrogel dressings. The incorporation of nanomaterials significantly impacts the mechanical properties of hydrogel dressings, as initially observed in their compressive and tensile strengths. The uniaxial compressive strength of the hydrogel dressings was evaluated, as shown in Fig. 2E. The endpoints of the curves indicate the compression fracture points. As the BTO@Au content increased from 0 mg/mL to 0.5 mg/mL, 1 mg/mL and 2 mg/mL, the uniaxial compressive stress at 60% strain for UNM10, UNM10/BA0.5, UNM10/BA1 and UNM10/BA2 increased were 0.014 MPa, 0.027 MPa, 0.043 MPa and 0.116 MPa, respectively. However, their compressive fracture strain decreased from 95% to 82%, 75% and 67%, respectively. This is attributed to the restriction of polymer chain movement by BTO@Au, which enhances the strength of the hydrogel dressings while increasing their brittleness. This trend is also evident in the tensile tests shown in Fig. 2F. As the BTO@Au content increased, the tensile strength at 50% strain for UNM10, UNM10/BA0.5, UNM10/BA1 and UNM10/BA2 increased from 4.24 kPa to 7.63 kPa, 10.95 kPa and 16.27 kPa, respectively.

Conductive hydrogels more effectively transmit biological regulatory signals, thereby promoting skin wound healing by regulating and enhancing cell adhesion, proliferation and migration [47]. Barium titanate exhibits piezoelectric properties, improves the hydrogel's conductivity and enhances the effective transmission of piezoelectric stimulation to wound tissues. The impact of BTO@Au content on the conductivity of hydrogel dressings was investigated using conductivity tests. As shown in Fig. 2G, the conductivity of UNM10 without BTO@Au was 9.35 × 10⁻² S/m, attributed to carboxylate ions on the gelatin molecular chains and the aqueous environment of the hydrogel dressing. The gold nanoparticles on BTO@Au are excellent electrical conductors, significantly enhancing the conductivity of the hydrogel. As the BTO@Au concentration increased from 0.5 mg/mL to 1 mg/mL and 2 mg/mL, the conductivity increased from 1.09 × 10⁻² S/m to 1.27 × 10⁻² S/m and 1.53 × 10⁻² S/m, respectively. These results indicate that varying the BTO@Au content can produce hydrogels with adjustable conductivity.

Biocompatibility is one of the most important requirements for dressing materials. The hemolysis activity of the hydrogels, the cytotoxicity of hydrogels for human umbilical vein endothelial cells (HUVECs) and human fibroblasts (HFBs), and the *in vivo* inflammatory response of the hydrogels were systematically evaluated. The hemolysis ratio of all groups was below 5%, indicating good blood compatibility (Fig. S5B and C). Besides, all four hydrogel dressings showed good cytocompatibility (cell viability greater than 80%), but a high concentration of BTO@Au in the hydrogel (UNM10/BA2) is detrimental to cell proliferation (Fig. S5A, D–G). Furthermore, all groups also showed mild inflammatory responses (Fig. S5H). The major organ histology, complete blood count, and serum biochemical parameters of mice showed no significant abnormalities after hydrogel implantation (Figs S6 and S7). All these results confirm that the UNMx/BAy hydrogel dressings exhibit excellent *in vitro* and *in vivo* biocompatibility.

### Photothermal and thermoresponsive properties of UNMx/BAy hydrogel

The incorporation of BTO@Au imparts excellent near-infrared (NIR) photothermal properties to the UNMx/BAy hydrogel dressings. The heat generated by the NIR photothermal effect serves as the heat source for the hydrogel's active contraction. Figure 2H demonstrates the impact of BTO@Au content on the photothermal performance of the hydrogel dressings under NIR irradiation at a fixed power density of 1.4 W/cm². After 600 seconds of NIR irradiation, the ΔT values for UNM10/BA0.5, UNM10/BA1 and UNM10/BA2 increased from 2.8°C to 8.3°C and 11.6°C as the BTO@Au content increased. UNM10 without BTO@Au showed no significant temperature change, indicating that the photothermal properties are attributed to BTO@Au. Additionally, the hydrogel dressings exhibit NIR power-density-dependent photothermal properties, where higher power densities result in larger ΔT after 600 seconds of irradiation. Notably, there was an inflection point in the photothermal curves between 260 and 300 seconds, where the temperature rise rate increased (Fig. 2I). This is attributed to the thermoresponsive contraction of the hydrogel, which increased the BTO@Au content per unit volume, thereby enhancing the photothermal conversion efficiency. A light power intensity of 2.0 W/cm² was selected for subsequent biological and animal experiments.

Thermoresponsive contraction was studied by immersing the hydrogel dressings in deionized water and heating them in a water bath to avoid shrinkage due to water loss. First, the surface contraction strain of the hydrogel dressings was measured at 37°C. As shown in Fig. 2J, after 30 minutes of incubation, the surface contraction strain decreased from 67.09% to 62.57%, 57.43% and 51.67% as the BTO@Au concentration increased from 0 to 0.5 mg/mL, 1 mg/mL and 2 mg/mL, respectively. The thermoresponsive contraction effect weakened with increasing BTO@Au content due to the restricted movement of polymer chains. Additionally, the thermoresponsive contraction effect was studied at 45°C. Before collecting surface contraction strain data, the hydrogel dressings were incubated at 37°C for 3 minutes to compare with the 37°C results. As shown in Fig. 2K, the surface contraction strain decreased from 76.68% to 70.18%, 65.21% and 61.88% as the BTO@Au concentration increased from 0 to 0.5 mg/mL, 1 mg/mL and 2 mg/mL. The overall trend of weakening thermoresponsive contraction with increasing BTO@Au content remained unchanged, but all samples showed higher surface contraction strains than those incubated only at 37°C. This is attributed to the designed mechanism of enhancing contraction efficiency upon heating and fixing contraction upon cooling.

The thermoresponsive contraction properties at the microlevel were studied using a field emission scanning electron microscope (SEM). As shown in Fig. 2L and M, at a BTO@Au concentration of 1 mg/mL, the internal pore diameter of UNM10/BA1 decreased from 57.0 μm at room temperature to 21.0 μm after 30 minutes of incubation at 37°C, confirming the thermoresponsive contraction properties. Increasing the incubation temperature to 45°C further reduced the internal pore diameter to 4.9 μm, confirming the effectiveness of the mechanism in enhancing thermoresponsive contraction efficiency. Additionally, we considered the potential effects of ultrasound-induced heat on the hydrogel's functionality and found no significant impact. The UNMx/BAy hydrogel exhibits good thermal stability and does not contain heat-sensitive substances like growth factors or exosomes, which prevents structural degradation. Furthermore, ultrasound heat did not affect the hydrogel's thermal shrinkage properties, as demonstrated by the similar wound contraction (90% for UNM10/BA1 + US + NIR + C-US and 80% for UNM10/BA1 + US + NIR) on day 5 (Fig. 4A and C). *In vitro* and *in vivo* experiments confirmed that the hydrogel maintains excellent antibacterial properties and regulates cell behavior under ultrasound stimulation. Overall, the heat generated by ultrasound does not compromise the hydrogel's versatility in wound healing.

### Sonopiezoelectric effect of BTO@Au and *in vitro* antibacterial performance of UNMx/BAy hydrogel

BTO is a widely used wide-bandgap ferroelectric semiconductor. In this study, gold nanoparticles were reduced and deposited on the surface of BTO nanocubes to form metal/semiconductor Schottky junctions. This modification promotes the separation and migration of electron-hole pairs by inducing band bending in BTO, thereby enhancing its piezoelectric catalytic performance [32]. The piezoelectric performance of BTO@Au was characterized using piezoresponse force microscopy (PFM). Figure S8A shows the phase-voltage curve of BTO@Au, where a 180° phase shift is observed when a bias voltage is applied. Meanwhile, the amplitude-voltage curve in Fig. S8B exhibits a clear butterfly loop characteristic with significant hysteresis. These results indicate that BTO@Au exhibits excellent piezoelectric properties.

Studies have shown that ultrasound can induce mechanical deformation and piezoelectric effects in BTO@Au [48], leading to the generation of electron-hole pairs, which in turn establish a strong built-in electric field on the material surface due to surface piezoelectric potential. Furthermore, the polarization charges and carriers generated by ultrasound catalysis interact with surrounding molecules (H₂O, O₂) through redox reactions, producing ROS. The singlet oxygen (¹O₂) generation capacity of BTO@Au was characterized using 1,3-diphenylisobenzofuran (DPBF) as an indicator, where ¹O₂ leads to a decrease in absorbance at 418 nm [49]. As shown in Fig. S8C, compared to the group without BTO@Au, 0.5 mg/mL BTO@Au significantly reduced the absorption peak at 418 nm, indicating the generation of ¹O₂. As the concentration of BTO@Au increased to 1 mg/mL and 2 mg/mL, the absorption peak at 418 nm further decreased, indicating a higher yield of ¹O₂ with increasing BTO@Au concentration. To explore the effect of ultrasound power density on ¹O₂ generation, we measured the absorption peak at 418 nm under different power densities. As shown in Fig. S8D, the absorption peak at 418 nm continuously decreased as the ultrasound power density increased from 0.3 W/cm² to 1.5 W/cm², indicating that the ¹O₂ generation capacity of BTO@Au increased with increasing ultrasound power density. The hydroxyl radical (·OH) generation capacity of BTO@Au was characterized using methyl violet (MV) as an indicator, where ·OH leads to a decrease in absorbance at 583 nm [49]. As shown in Fig. S8E and F, similar to ¹O₂, both the increase in BTO@Au concentration and ultrasound power density resulted in an enhanced ·OH generation capacity.

**Figure 3.**
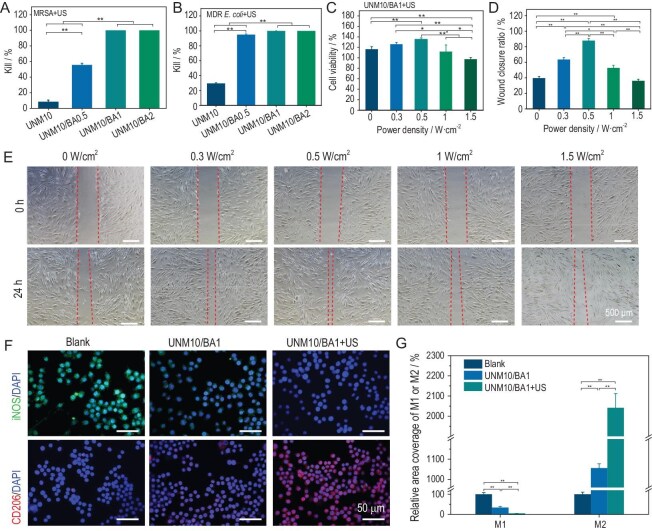
Sonopiezoelectric-effect-mediated regulatory effects of UNMx/BAy. (A and B) Bactericidal ratios of hydrogels against MRSA (A) and MDR *E. coli* (B) with 1.5 W/cm² ultrasound treatment. (C) Cell viability under exogenous ultrasound-mediated UNMx/BAy treatment. (D and E) Wound closure ratio (D) and representative images (E) of cell scratches at 24 hours post-treatment with hydrogels mediated by ultrasound at different power densities. (F and G) Representative images (F) and relative expression levels (G) of M1 macrophages and M2 macrophages under different treatment conditions. *P < 0.05, **P < 0.01.

ROS exhibit excellent bactericidal properties by peroxidizing polyunsaturated phospholipids in bacterial membranes, thereby disrupting their selective permeability [50]. In this study, we used high-power-density (1.5 W/cm²) external ultrasound stimulation to induce high levels of ROS in UNMx/BAy hydrogel dressings for antibacterial purposes. MRSA and multidrug-resistant *Escherichia coli* (MDR *E. coli*) were selected as representative Gram-positive and Gram-negative bacteria, respectively, for *in vitro* antibacterial experiments. To account for heat generation affecting bacterial activity under ultrasound stimulation, the plates containing bacterial solution and materials were pre-cooled on ice for 5 minutes before ultrasound treatment. As shown in Fig. S8G–J, the hydrogel dressings exhibited no significant antibacterial activity against MRSA and MDR *E. coli* without ultrasound treatment. In contrast, ultrasound-treated hydrogel dressings showed varying degrees of antibacterial activity. As shown in Fig. S8K and L, and Fig. 3A and B, the sonopiezoelectric antibacterial ratios of the hydrogel dressings increased with the concentration of BTO@Au. Notably, UNM10/BA1 and UNM10/BA2 exhibited more than 99.9% antibacterial efficacy against both MRSA and MDR *E. coli*, demonstrating excellent antibacterial properties. The relatively weak antibacterial activity of UNM10 without BTO@Au (MRSA killing ratio <9%; MDR *E. coli* killing ratio <30%) might be attributed to mechanical damage to bacterial cells caused by ultrasonic cavitation [51].

Finally, the effect of ultrasound stimulation on the morphology of bacteria treated with hydrogel dressings was observed using SEM. As shown in Fig. S9, the MRSA and MDR *E. coli* in the blank group and UNM10 group retained their normal spherical and rod-shaped morphologies, respectively. The slight deformation observed can be attributed to thermal and mechanical damage from the ultrasonic cavitation effect and the initial membrane stress induced by the interaction between the material and bacteria [51,52]. For UNM10/BA0.5, UNM10/BA1 and UNM10/BA2 containing BTO@Au, obvious wrinkles and significant collapse were observed on the surface of some bacteria under ultrasound stimulation. This indicates that the hydrogel dressings, under ultrasound stimulation, can disrupt the bacterial cell membrane by releasing ROS, thereby compromising the integrity of bacterial morphology and achieving antibacterial effects. Based on these results, UNM10/BA1 was selected for further study.

### Positive effects of sonopiezoelectric-effect-mediated UNMx/BAy on the behavior of HFB cells and macrophages

To further investigate the impact of sonopiezoelectric effect on cell viability, HFB cells were stimulated with ultrasound at different power densities (0.3 W/cm², 0.5 W/cm², 1 W/cm² and 1.5 W/cm²) for 1 minute, and cell viability was assessed after 24 hours, with the 0 W/cm² group used as a control. As shown in Fig. S8M, simple ultrasound stimulation did not promote cell proliferation, and cell viability showed a slight decreasing trend with increasing ultrasound power density. However, when the UNM10/BA1 hydrogel dressing was added, ultrasound at power densities of 0.3 W/cm² and 0.5 W/cm² promoted cell proliferation, with cell viability reaching 126% and 135%, respectively (Fig. 3C). Compared to the control group, cell viability decreased at ultrasound power densities of 1 W/cm² and 1.5 W/cm². To further assess the impact of different ultrasound power densities on cell movement, a scratch assay was performed on HFB cells using the same method. Figure 3E shows that under co-culture with the UNM10/BA1 hydrogel, ultrasound at power densities of 0.3 W/cm² and 0.5 W/cm² exhibited superior cell migration effects. Quantitative analysis showed that ultrasound treatment at 0.5 W/cm² resulted in an 88% wound closure ratio, significantly higher than other groups (Fig. 3D). These results indicate that the cell proliferation and migration-promoting effects of the UNM10/BA1 hydrogel are most pronounced at an ultrasound power density of 0.5 W/cm², likely due to the low-level ROS generated by the piezoelectric effect of ultrasound and the formation of a localized micro electric field [34,53].

The influence of the localized micro electric field generated by the sonopiezoelectric effect on macrophage differentiation was further studied. As shown in Fig. 3F and G, without ultrasound treatment, UNM10/BA1 inhibited macrophage differentiation towards the M1 phenotype and promoted differentiation towards the M2 phenotype, likely due to the excellent conductivity of the piezoelectric effect of the UNM10/BA1 hydrogel. Studies have shown that piezoelectric hydrogels can regulate macrophage M2 polarization by generating a piezoelectric microenvironment [54]. After ultrasound treatment, the functional effects of UNM10/BA1 became more pronounced, as the ultrasound stimulation enhanced the piezoelectric effect of the hydrogel, forming a localized micro electric field and subsequently regulating macrophage behavior.

**Figure 4.**
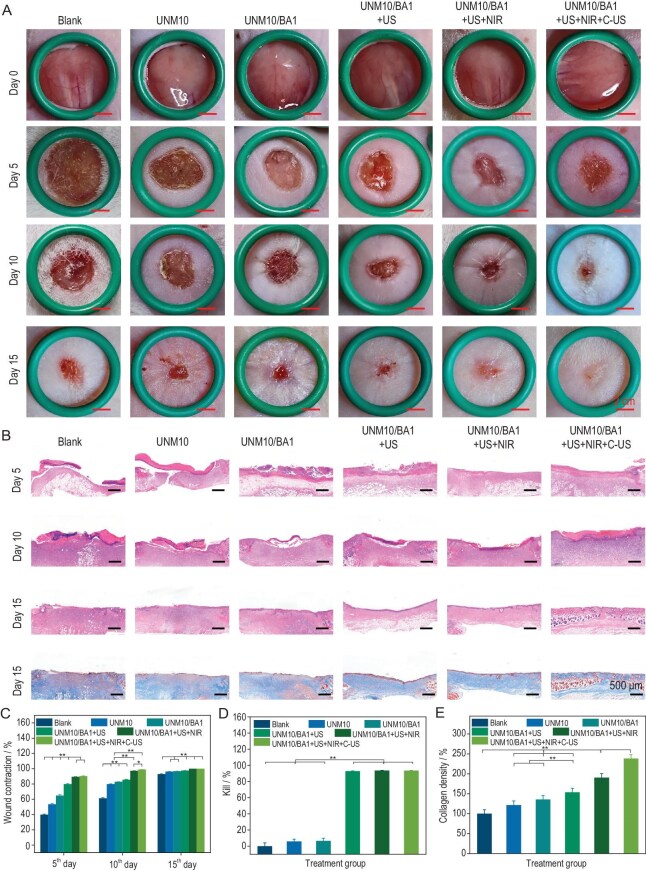
(A) Representative images of full-thickness skin wounds on the neck of rats on days 5, 10, 15 and the initial day of treatment. (B) Representative H&E-stained images of wounds on days 5, 10 and 15, and Masson's trichrome-stained images on day 15 for different treatment groups. (C) Wound closure ratios on days 5, 10 and 15 for different treatment groups. (D) *In vivo* antibacterial ratios on day 5 for different treatment groups. (E) Collagen density at the wound site on day 15 for different treatment groups. *P < 0.05, **P < 0.01.

Additionally, our results demonstrate that the ultrasonic piezoelectric hydrogel significantly enhances epidermal cell (HaCaT) migration. *In vitro* scratch assays (Figs S10 and S11) revealed a 6.2-fold increase in keratinocyte migration speed in the UNM10/BA1 + US + NIR + C-US-treated hydrogel group compared to the control (*P* < 0.01). This may be attributed to US-triggered piezoelectric potentials, which induce electrotaxis and activate voltage-gated Ca²⁺ channels [55,56], promoting cytoskeletal reorganization via RhoA/ROCK signaling pathway [52]. Furthermore, *in vivo* data (Fig. 4C) revealed accelerated epidermal migration (50% faster wound closure) in the treatment groups, consistent with the observed re-epithelialization outcomes.

### Sonopiezoelectric-effect-mediated promotion of neck-wound healing in MRSA-infected mice by UNMx/BAy

The efficiency of wound healing was evaluated using a full-thickness skin defect model on the necks of MRSA-infected mice. This study investigated how UNMx/BAy hydrogel dressings enhance wound healing through active thermally induced contraction and sonopiezoelectric effect. The optimized UNM10/BA1 was selected as the primary treatment group, with UNM10 and untreated blank groups serving as controls. Additionally, three experimental groups were established based on different modes of exogenous stimulation. The UNM10/BA1 + US group involved treatment with UNM10/BA1 and the application of single exogenous high power ultrasound stimulation at a power density of 1.5 W/cm² for 5 minutes, aiming to generate high levels of ROS to eliminate bacteria at the wound site. The UNM10/BA1 + US + NIR group involved, in addition to the UNM10/BA1 + US treatment, the application of exogenous 808 nm near-infrared irradiation at a power density of 2.0 W/cm², intended to raise the temperature of UNM10/BA1 to around 45°C, thereby promoting wound closure through active thermally induced contraction by pulling the wound edges together. The UNM10/BA1 + US + NIR + C-US group involved, in addition to the UNM10/BA1 + US + NIR treatment, daily exogenous low power ultrasound stimulation at a power density of 0.5 W/cm² for 20 minutes during the treatment period, aiming to continuously promote wound healing through the formation of a localized micro electric field and low-level ROS by ultrasound.

Wound closure in mice was first studied. As shown in Fig. 4A, after creating a circular full-thickness skin defect with an 8 mm diameter on the necks of the mice using a tissue biopsy punch, various treatments were applied to the wound. Overall, the UNM10 group demonstrated some therapeutic effects compared to the blank group, likely due to the hydrogel dressing's sealing properties and the gelatin components’ ability to promote cell adhesion and proliferation. Among the four additional treatment groups, both the UNM10/BA1 + US + NIR and UNM10/BA1 + US + NIR + C-US groups exhibited significantly stronger wound healing effects. As shown in Fig. 4C, a quantitative analysis of wound healing ratios was conducted on days 5, 10 and 15 of treatment. On day 5, the wound healing ratio in the UNM10/BA1 group (65%) was significantly higher than that in the UNM10 group (53%), which may be attributed to the higher conductivity conferred by BTO@Au in the hydrogel dressing, facilitating the transmission of biological signals that promote the migration of immune cells and other cells [57]. In addition, the movement of the neck wound may also generate localized micro electric field stimulation. The higher wound healing ratio observed in the UNM10/BA1 + US group (80%) may be attributed to the cumulative antibacterial effect. By utilizing the inherent antibacterial properties of nanoparticles, ultrasound stimulation further enhances their effectiveness, resulting in a more pronounced antibacterial impact. Under the photothermal effect of near-infrared light, the wound healing ratios in the UNM10/BA1 + US + NIR and UNM10/BA1 + US + NIR + C-US groups reached 90%, as the hydrogel's active thermally induced contraction not only assisted in the rapid reduction of the wound area macroscopically but also induced fibroblast proliferation and migration through biomechanical stimulation microscopically [19]. On day 10, the wound healing ratio in the UNM10/BA1 + US + NIR + C-US group was significantly higher than that in the UNM10/BA1 + US + NIR group (*P* < 0.05). This improvement may be attributed to the continuous generation of low-level ROS and a localized micro electric field induced by low-power ultrasound, along with micro piezoelectric stimulation generated by the deformation of the piezoelectric hydrogel during wound movement caused by exercise.

Histomorphometric analysis of hematoxylin and eosin (H&E)-stained sections (day 15) showed that the UNM10/BA1-US + NIR + C-US group achieved near-normal epidermal thickness (28.0 ± 1.3 μm vs. 26.1 ± 1.0 μm in unwounded skin, *P* > 0.05), whereas the blank group exhibited hyperproliferative epithelium (153.6 ± 7.9 μm, *P* < 0.01, Fig. S12). The gap between dermal-epidermal junctions was analyzed to reflect regeneration quality (Fig. S13). The treatment group displayed a smaller gap of 3.95 ± 0.74 μm, significantly less than the blank group (51.72 ± 5.75 μm, *P* < 0.01). The reason may be attributed to the fact that low-level ROS and localized electrical stimulation promote cell migration at the wound edges, aiding in the filling of gaps [34,53].

Bacteria at the wound site were collected and counted on day 5 to assess the antibacterial effect of the hydrogel dressing in a physiological environment. As shown in Figs 4D and S14, UNM10 and UNM10/BA1 without ultrasound treatment did not exhibit significant antibacterial properties, with an MRSA-killing ratio of only ∼6%. The MRSA-killing ratio in the ultrasound-treated UNM10/BA1 + US group exceeded 92%, indicating that the sonopiezoelectric effect can provide excellent antibacterial effects in a physiological environment via generating high level of ROS. Additionally, we verified the impact of the selected photothermal power density on the antibacterial effect, and the results showed that the selected near-infrared photothermal treatment did not significantly affect the antibacterial results.

H&E staining and Masson's trichrome staining were used to further evaluate wound healing. As shown in the H&E-stained images in Fig. 4B, on day 5 of treatment, none of the groups formed complete epithelial tissue, and the wound healing process was still in the inflammatory phase of a chronic infected wound. However, compared to the blank, UNM10 and UNM10/BA1 groups, the UNM10/BA1 + US, UNM10/BA1 + US + NIR and UNM10/BA1 + US + NIR + C-US groups showed less inflammatory infiltration, with significantly fewer inflammatory cells in the wound area. The results revealed early collagen deposition in the UNM10/BA1 + US + NIR + C-US groups on day 5 (Fig. S15A and C), with a 5.2-fold higher collagen density compared to the control (*P* < 0.01), suggesting accelerated extracellular matrix (ECM) remodeling. On day 10, with the assistance of a hydrogel dressing with active, thermally-induced contraction and continuous low-power ultrasound stimulation, only the UNM10/BA1 + US + NIR and UNM10/BA1 + US + NIR + C-US groups formed relatively complete epithelial tissue. Meanwhile, collagen maturation was improved, as evidenced by a higher collagen ratio in the UNM10/BA1 + US + NIR + C-US group on day 10 (3.8-fold higher than the control, *P* < 0.01), indicating more advanced tissue repair (Fig. S15B and D).

The collagen density in the blank and UNM10 groups was significantly lower than in the other treatment groups on day 15. Further quantitative analysis (Fig. 4E) revealed that the relative collagen density in the UNM10/BA1 + US + NIR + C-US group (238%) was significantly higher than in the other groups, demonstrating the best promotion of collagen deposition. On day 15, re-epithelialization was not completed in the blank and UNM10 groups. The UNM10/BA1 + US, UNM10/BA1 + US + NIR and UNM10/BA1 + US + NIR + C-US groups formed complete and smooth epithelial tissue, particularly in the UNM10/BA1 + US + NIR + C-US group, which developed relatively mature hair follicles, indicating better functionalization of the newly formed skin tissue. Our data suggest that low-power ultrasound promotes hair follicle regeneration through two synergistic mechanisms: a piezoelectric field generated by BTO@Au, which activates phosphoinositide 3-kinase (PI3K)/protein kinase B (AKT) pathways (Fig. 6D), and mechanical stimulation from the contracted hydrogel, which enhances mechanotransduction pathways like integrin-focal adhesion kinase (FAK) (Fig. 6D). These pathways are known to be crucial in regulating various aspects of cellular responses to mechanical stimuli, and previous studies have demonstrated a link between mechanical stimulation and hair follicle neogenesis [58].

In summary, the results of wound healing ratio, H&E staining and Masson staining studies indicate that the UNM10/BA1 + US + NIR + C-US group achieves optimal therapeutic effects for chronic MRSA-infected skin wounds. This is achieved through wound closure via thermal contraction, high-power-density ultrasound stimulation generating high levels of ROS for efficient antibacterial purposes, low-power-density ultrasound stimulation generating low levels of ROS, and sustained local micro electric field formation. These effects collectively help to eliminate wound bacteria, promote wound contraction and healing, reduce inflammatory infiltration, enhance skin re-epithelialization and increase collagen deposition.

### *In vivo* expression levels of ROS, TNF-α and VEGF, and blood perfusion statistics in different treatment groups during wound healing

**Figure 5.**
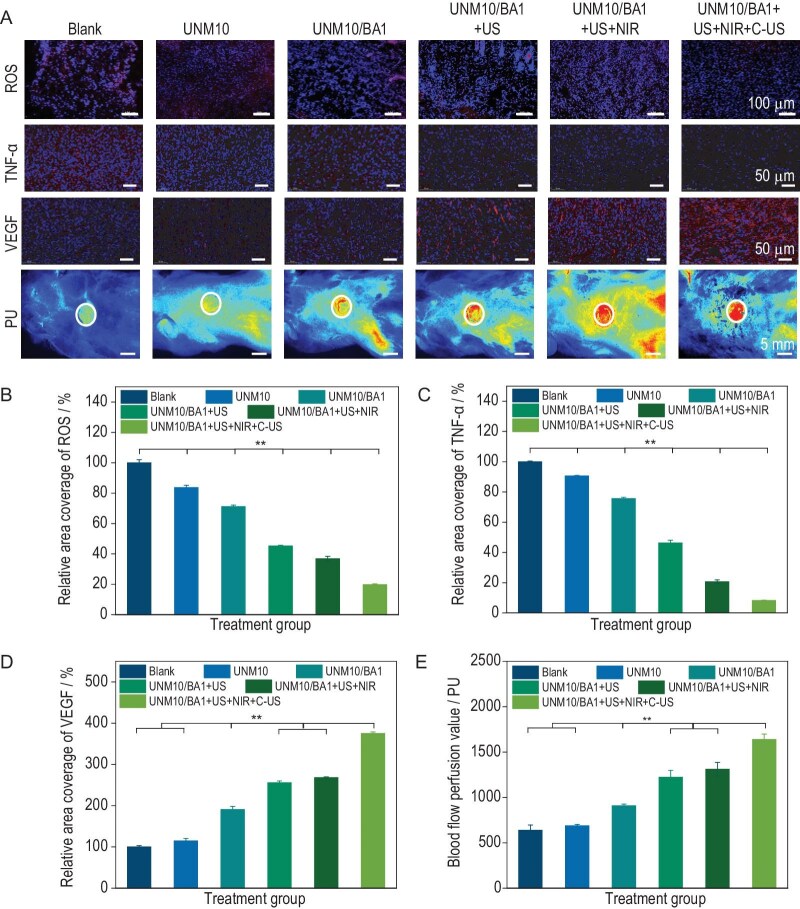
(A) Representative images of *in vivo* ROS staining on day 5, TNF-α immunofluorescence staining on day 5, VEGF immunofluorescence staining on day 15 and laser Doppler imaging on day 5. (B) Relative ROS content *in vivo* on day 5. (C) Relative TNF-α expression on day 5. (D) Relative VEGF expression on day 15. (E) Average blood flow perfusion in the wound area on day 5. *P < 0.05, **P < 0.01.

The inflammatory phase is a crucial aspect of skin-wound healing, and bacterial infection can lead to a persistent inflammatory state in the wound area, thereby hindering the healing process. The regulation of wound inflammation by hydrogel dressings was assessed by *in vivo* ROS staining of the newly formed tissue. As shown in Fig. 5A, on day 5 of treatment, ROS levels in the wound tissue were evaluated using dihydroethidium (DHE) probes. The progressive treatment strategies led to lower *in vivo* ROS levels. Compared to the blank, UNM10 and UNM10/BA1 groups, the red fluorescence in the UNM10/BA1 + US, UNM10/BA1 + US + NIR and UNM10/BA1 + US + NIR + C-US groups was significantly diminished, indicating a substantial reduction in *in vivo* ROS levels. The quantitative analysis shown in Fig. 5B also reflected the same trend.

To quantify the hydrogel's immunomodulatory effects, TNF-α expression was analyzed via immunofluorescence on day 5 (Fig. 5A and C) and day 10 (Fig. S16A). In the early inflammation phase (day 5), using the blank group as a control, the relative expression levels of TNF-α in the UNM10 and UNM10/BA1 groups exceeded 75%. The UNM10/BA1 + US + NIR + C-US group showed over 90% reduction in TNF-α versus control (*P* < 0.01), indicating rapid suppression of acute inflammation (Fig. 5C). In contrast, the relative expression levels in the UNM10/BA1 + US and UNM10/BA1 + US + NIR groups decreased to 46% and 40%, respectively. In the transition to the proliferation phase (day 10), TNF-α levels in the treatment group were reduced by 93% compared to the control (*P* < 0.01), confirming sustained anti-inflammatory activity (Fig. S16A and C). This reduction is associated with the antibacterial effect of high levels of ROS released by hydrogel dressings under high-power-density ultrasound stimulation, which reduces the infection state of the wound area. The UNM10/BA1 + US + NIR + C-US group exhibited the lowest inflammatory state, with a relative expression level of only 8.1%, attributable to the micro electric field generated by continuous low-power-density ultrasound treatment, which may polarize macrophage phenotypes toward anti-inflammatory M2 state (Fig. 3F). This shift reduces inflammatory cytokines (e.g. TNF-α, IL-6) while elevating reparative factors (IL-10, TGF-β), as demonstrated in macrophage mechanobiology studies [59]. Previous studies have also shown that low-level electrical stimulation can produce effective anti-inflammatory responses, reducing inflammation caused by various stimuli [26].

Adequate oxygen and nutrients are essential for cell proliferation, migration and cytokine transport during wound healing, necessitating the development of a well-established vascular network in the wound area. The angiogenic capacity of each treatment group was evaluated by immunofluorescence staining for vascular endothelial growth factor (VEGF). As shown in Fig. 5A and D, the relative expression of VEGF in the UNM10/BA1 group increased to 190%, compared to 100% in the blank group and 115% in the UNM10 group. This increase is associated with the piezoelectric properties of UNM10/BA1, where the micro electric field stimulation generated by the movement of the wound helps regulate the transmission of biological signals in the wound area. Due to the antibacterial effect of high levels of ROS generated by high-power-density ultrasound stimulation, the healing process improved, with VEGF expression levels increasing to 256% and 268% in the UNM10/BA1 + US and UNM10/BA1 + US + NIR groups, respectively. The UNM10/BA1 + US + NIR + C-US group demonstrated the highest angiogenic capacity, with VEGF expression reaching 375%. Besides, immunofluorescence co-staining for CD31 (endothelial cells) and α-SMA (smooth muscle cell) on day 10 revealed enhanced neovascularization in treatment groups. The UNM10/BA1 + US + NIR + C-US group exhibited a 7-fold increase in mature vessels (CD31⁺/α-SMA⁺ structures) compared to the control (*P* < 0.01, Fig. S16B and D). This indicates that the continuous low levels of ROS released by low-power-density ultrasound stimulation and the sustained formation of a localized micro electric field effectively promote angiogenesis in the wound area. Additionally, the photothermal effect of gold nanoparticles absorbing NIR light enhances local blood circulation, improves oxygenation and activates the HIF-1α/VEGF axis, further supporting vascular formation and promoting wound healing [60]. These combined effects significantly accelerate vascular regeneration in chronic wounds.

Adequate blood supply is a key factor for effective wound healing. To further assess vascular function in the wound area during the early stages of wound healing, laser Doppler imaging was used to study blood flow perfusion in the wound area on day 5. Blood perfusion was evaluated using a laser speckle flow imaging system with a near-infrared laser at a wavelength of 785 nm, based on the generated speckle pattern and its dynamic changes. As shown in Fig. 5A, the representative laser Doppler images indicate a gradient of blood perfusion from low to high levels, corresponding to the color transition observed in the images. Except for the UNM10 group, more red regions, indicating higher blood perfusion, were observed in the other treatment groups. With the progressive treatment strategies, more blood perfusion was observed in the wound area, with the UNM10/BA1 + US + NIR + C-US group showing the most significant treatment effect. As shown in Fig. 5E, the blood perfusion values calculated using the software of the laser speckle flow imaging system were 1223 PU, 1331 PU and 1640 PU for the UNM10/BA1 + US, UNM10/BA1 + US + NIR and UNM10/BA1 + US + NIR + C-US groups, respectively, significantly higher than the 638 PU of the blank group. This improvement in the wound healing environment is attributed to the elimination of bacteria in the wound area by high levels of ROS. The UNM10/BA1 + US + NIR + C-US group had significantly higher blood perfusion than the other treatment groups, which is related to the continuous low-power-density ultrasound stimulation of the hydrogel dressing, releasing low levels of ROS and forming a localized micro electric field that promotes angiogenesis. These results are consistent with VEGF expression levels in each group.

In conclusion, the UNM10/BA1 + US + NIR + C-US group, with its on-demand sonopiezoelectric effect and thermally induced contraction to promote wound closure, effectively enhances the healing of chronically infected wounds. This is achieved through promoting wound closure, collagen deposition, proliferation and migration of skin-repair-related functional cells, regulating inflammation and promoting angiogenesis. Sonopiezoelectric therapy enhances skin-wound healing through various mechanisms. High levels of ROS oxidize lipids in bacterial cell membranes, disrupting their structure and causing leakage of intracellular proteins, ultimately eliminating bacteria [32]. Low levels of ROS can induce platelet aggregation and release platelet-derived growth factor (PDGF), promoting the proliferation and migration of functional cells involved in skin repair [31]. Additionally, low levels of ROS facilitate redox signaling and, together with the endogenous electric field generated by the piezoelectric effect, promote the proliferation of fibroblasts and keratinocytes, aiding in the repair of chronic infected wounds.

### Mechanistic study of the sonopiezoelectric-effect-mediated promotion of neck-wound healing by UNMx/BAy in MRSA-infected mice

**Figure 6.**
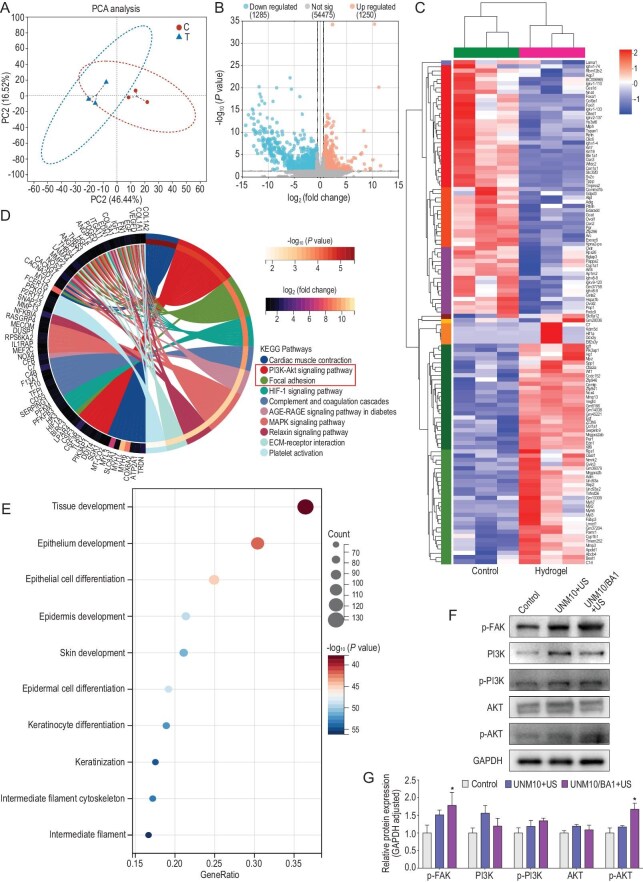
Sonopiezoelectric-effect-mediated hydrogel treatment of chronic infected wounds: RNA-seq analysis. (A) Principal component analysis (PCA) of the transcriptomic profiles between the control group and the UNM10/BA1 + US + NIR + C-US treated group, with each data point representing a sample. (B) Volcano plot showing up-regulated (1250) and down-regulated (1285) genes. (C) Heatmap showing clustering analysis of differentially expressed genes after UNM10/BA1 + US + NIR + C-US treatment. (D and E) Kyoto Encyclopedia of Genes and Genomes (KEGG) and gene ontology (GO) enrichment analysis of up-regulated genes after UNM10/BA1 + US + NIR + C-US treatment. (F) p-FAK and PI3K/AKT signaling pathway protein expression in HFB cells among the control group, UNM10 + US and the UNM10/BA1 + US treated group. (G) Quantitative analysis of protein expression levels.

To further investigate the mechanism by which UNM10/BA1 hydrogel promotes wound healing under ultrasound, wound tissue samples were collected on day 10 for RNA sequencing (RNA-seq), using Tegaderm film-treated neck wounds as controls. Principal component analysis (PCA) revealed distinct differences between the transcriptomes of the control and UNM10/BA1 + US + NIR + C-US treated groups (Fig. 6A). As shown in the volcano plot, a total of 2535 differentially expressed genes (DEGs) were identified, including 1250 up-regulated genes and 1285 downregulated genes (fold change ≥ 1.5, *P* < 0.05) (Fig. 6B).

Enrichment analysis of the DEGs was performed for both up-regulated and downregulated gene sets, with heatmap analysis showing the clustering of differential genes in the wound tissue of the control and UNM10/BA1 + US + NIR + C-US treated groups (Fig. 6C). Specifically, the hydrogel treatment group significantly up-regulated genes related to angiogenesis and wound healing, including Hif1α, Egf, Vegfd, Hgf and Spp1 (Fig. 6C). The HIF-1α (hypoxia-inducible factor-1α) gene plays a key role in wound healing; studies have shown that slow healing in chronically infected wounds is associated with decreased HIF-1α expression. In chronically infected wounds, inflammation causes endothelial dysfunction, inhibiting HIF-1α expression [61]. Thus, the increased HIF-1α expression in the UNM10/BA1 + US + NIR + C-US treatment group aids in healing infected wounds. Additionally, Spp1 (osteopontin, OPN), produced by macrophages, acts as a pro-repair factor during wound healing. SPP1 macrophages promote vascular stabilization and wound healing by releasing growth factors and cytokines such as VEGF [62] and transforming growth factor-β1 (TGF-β1) [63]. Under hypoxic conditions, SPP1 macrophages generate factors that induce and accelerate angiogenesis, aiding re-epithelialization, granulation tissue formation and new extracellular matrix production, thus promoting wound healing [64]. These findings confirm that the UNM10/BA1 + US + NIR + C-US treatment group creates a microenvironment conducive to chronic wound healing, enhancing repair.

Furthermore, Kyoto Encyclopedia of Genes and Genomes (KEGG) pathway analysis of up-regulated genes indicated that the UNM10/BA1 + US + NIR + C-US treatment group promoted enrichment in pathways such as cardiac muscle contraction, PI3K-Akt, focal adhesion, HIF-1 and MAPK signaling, which enhance tissue regeneration (Fig. 6D). Notably, the shrinking hydrogel promoted the expression of genes related to focal adhesion, potentially linked to its contractility and piezoelectric effects. FAK is a critical component of the focal adhesion signaling pathway. The shrinking hydrogel induces wound contraction, generating biomechanical forces that are transmitted to the cell interior via integrins on the cell surface. The interaction between integrins and the extracellular matrix (ECM) is altered under mechanical force, leading to integrin clustering and conformational changes that activate FAK. The activation of FAK promotes cell proliferation through the MAPK/ERK and PI3K/AKT pathways, increasing the cell population in the wound area and accelerating new tissue formation [65]. Studies have also shown that composite piezoelectric materials significantly enhance bone marrow mesenchymal stem cell adhesion and spreading, reflected in the increased number and area of mature focal adhesions [66]. During chronic wound healing, cell migration is crucial for wound coverage and closure. Additionally, gene ontology (GO) analysis revealed that 1259 up-regulated genes were concentrated in the positive regulation of tissue development, epidermis development and epithelial cell differentiation (Fig. 6E).

Given that the sonopiezoelectric effect of nanoparticles released from the shrinking hydrogel might promote chronic wound healing by activating the FAK and AKT signaling pathway, we tested this hypothesis by extracting proteins from HFB cells treated with control, UNM10 + US and UNM10/BA1 + US. Western blot analysis was performed to assess the expression levels of p-FAK, PI3K, p-PI3K, AKT and p-AKT proteins (Fig. 6F and G). The results indicated that only when the hydrogel contained nanoparticles did ultrasound treatment significantly increase the expression of p-FAK and p-AKT in cells (Fig. 6F), while the up-regulation of p-FAK and p-PI3K in the UNM10 + US treatment group was not statistically significant compared to the control.

During wound healing, cell proliferation, migration and survival are prerequisites for successful healing. Studies have shown that p-FAK promotes the activation of p-AKT through its interaction with PI3K, and p-AKT further enhances cell survival and proliferation, forming a positive feedback loop that strengthens cellular responses to environmental signals [67]. p-Akt can promote angiogenesis, and one of the key reasons for the difficulty in healing infectious chronic wounds is inflammation-induced vascular damage and reduced neovascularization capacity. The activation of p-AKT protein could promote endothelial cell proliferation and migration, increasing vascular density and improving blood supply to chronic wounds, thereby facilitating wound healing [54]. Additionally, up-regulation of p-AKT can reduce the inflammatory response by regulating the expression of inflammation-related factors such as TNF-α, interleukin-1 beta (IL-1β) and interleukin-10 (IL-10) [68], which is beneficial for wound healing. Meanwhile, p-AKT is a critical survival signal that inhibits apoptosis-related proteins, such as BAD and Caspase-9, thereby increasing cell survival under adverse conditions [69]. Furthermore, up-regulation of the p-AKT gene can promote the migration and proliferation of keratinocytes and fibroblasts, thereby reducing the damaged area and promoting wound healing [70]. Therefore, UNM10/BA1 hydrogel, through contraction and sonopiezoelectric effect, activates the FAK and AKT signaling pathways, ameliorating the adverse microenvironment of the wound, and accelerating wound healing.

Other studies have shown that the transcription factor SPP1 can promote the activation of the FAK and AKT signaling pathway [69,71,72], and the up-regulation of the SPP1 gene in the UNM10/BA1 + US + NIR + C-US treatment group might also contribute to the activation of the FAK and AKT signaling pathway (Fig. 6C). However, it should be noted that the specific mechanisms of SPP1 and the FAK and AKT pathway with regard to the healing process of chronic wounds are not yet fully elucidated and require further research.

While our study highlights the potential of dual-network hydrogels in dynamic chronic wound management, several challenges remain before real-world application can occur. First, long-term stability and biocompatibility under clinical conditions (e.g. gelatin derivatives and BTO@Au) need further evaluation, especially in human patients. Future studies will include more comprehensive *in vivo* models, such as pig skin injury models, to assess long-term effects and potential adverse reactions. Second, optimizing the hydrogel's mechanical properties to withstand real-world stresses is crucial, particularly considering the biomechanical differences between animal models and human patients. Improvements are needed in durability, flexibility and functionality under dynamic conditions, with efficacy validated in diverse clinical scenarios (e.g. diabetic ulcers and burn wounds). Third, scaling up hydrogel production presents significant challenges in terms of cost-effectiveness and reproducibility, especially with piezoelectric materials like BTO@Au. Exploring low-cost, scalable alternatives with similar ultrasonic properties is essential for broader clinical use. Fourth, careful control of the dressing contraction rate is necessary to prevent scarring, and future studies should focus on optimizing this rate to match normal skin growth. Finally, precise control of ROS release and electrical stimulation based on wound healing stages is critical for clinical translation. Further studies are needed to optimize ROS generation, avoiding harmful effects in later healing stages. In conclusion, to advance this hydrogel toward clinical application, we will focus on enhancing its stability, scalability and control mechanisms. We envision developing key devices, including a smartphone-controlled wearable stimulation kit and a smart bandage with sensors, to enable autonomous stimulation triggering, ensuring adaptive wound healing management.

## CONCLUSION

We designed and fabricated an ultrasound/NIR dual-response double-network tough hydrogel dressing with on-demand thermal contraction and sonopiezoelectric effect, which combined on-demand wound closure in the initial stage with a transition from antibacterial and anti-inflammatory actions to subsequent healing stages, to improve the whole stage management of dynamic infected chronic-wound repair. The first network of the hydrogel is composed of UPy-GU, forming a physical network structure via hydrogen bonding. The second network consists of NIPAM and GMA, polymerized into a chemical network structure. The GU-based network possesses two key properties: firstly, the hydrogel dressing's strength is enhanced by the breaking of hydrogen bonds under external load; secondly, the dynamic nature of physical bonds imparts a degree of toughness to the hydrogel dressing. Additionally, the hydrogen bonds between GUs can gradually break with rising temperature and reform upon cooling, enabling a mechanism for temperature-induced contraction and fixation. Experimental results indicate that this mechanism significantly enhances the thermally induced contraction rate of the hydrogel dressing. In the second network of the hydrogel, PNIPAM serves as the source of hydrogel contraction, while GMA, acting as a macromolecular crosslinker, further enhances the toughness of the hydrogel dressing. BTO@Au, with its piezoelectric effect, serves as both a conductive component and a sonopiezoelectric functional component. *In vitro* biological experiments demonstrated that the hydrogel dressing could release high levels of ROS to eliminate bacteria at the wound site when exposed to ultrasound at a power density of 1.5 W/cm² for 5 minutes. Under ultrasound at a power density of 0.5 W/cm² for 1 minute, the hydrogel dressing released low levels of ROS and formed a localized micro electric field, significantly promoting human fibroblast proliferation and migration, while the piezoelectric effect of nanoparticles promoted macrophage M2 polarization. Overall, the treatment strategy offered by UNMx/BAy hydrogel dressings consists of three stages. First, high levels of ROS were released by the hydrogel dressing under exogenous high-power-density ultrasound stimulation to efficiently remove bacteria from the wound. Next, the dressing was irradiated with near-infrared light to induce volume contraction by collapse of PNIPAM under hot conditions, thus pulling the wound edge closed. Finally, under the stimulation of low-power-density ultrasound, low levels of ROS were released for a long time to further promote repair by promoting cell migration, collagen deposition, angiogenesis and inflammation regulation. Using a full-thickness skin defect model in the necks of MRSA-infected mice, the UNM10/BA1 + US + NIR + C-US group effectively promoted the healing of chronically infected wounds by eliminating bacteria, promoting collagen deposition, regulating inflammation, increasing blood perfusion in the wound area, enhancing fibroblast migration, inducing angiogenesis and achieving active thermally induced contraction to pull the wound edges together, which significantly activated the FAK and AKT signaling pathways in wound healing, demonstrating great potential for clinical application.

## METHODS

Experimental methods are available in the Supplementary Data. All animal experiments were approved by the Animal Research Committee of Xi’an Jiaotong University.

## Supplementary Material

nwaf118_Supplemental_File
