# Supplementary material for: Piezoelectric dual-network tough hydrogel with on-demand thermal contraction and sonopiezoelectric effect for promoting infected-joint-skin-wound healing via FAK and AKT signaling pathways
Source: Natl Sci Rev. 2025 Mar 29;12(5):nwaf118. doi: 10.1093/nsr/nwaf118 (PMC12042750; doi:10.1093/nsr/nwaf118)
Supplement: nwaf118_Supplemental_File [file nwaf118_supplemental_file.pdf]

Piezoelectric dual-network tough hydrogel with on demand thermal contraction and sonopiezoelectric effect for promoting infected joint skin wound healing via FAK and AKT signaling pathways

Jinlong Luo[a,†], Zhen Liang[b,†], Xin Zhao[a,*], Shengfei Huang[a], Yanan Gu[b], Zexing Deng[c], Jing Ye[a], Xingmei Cai[a], Yong Han[a,d] and Baolin Guo[a,d,e,*]

[a]State Key Laboratory for Mechanical Behavior of Materials, and Frontier Institute of Science and Technology, Xi'an Jiaotong University, Xi'an 710049, China;

[b]Department of Plastic Surgery, Xijing Hospital, Fourth Military Medical University, Xi'an 710032, China;

[c]College of Materials Science and Engineering, Xi'an University of Science and Technology, Xi'an 710054, China;

[d]Department of Orthopaedics, The First Affiliated Hospital of Xi'an Jiaotong University, Xi'an 710061, China;

[e]Department of Dermatology, The Second Affiliated Hospital of Xi'an Jiaotong University, Xi'an 710004 China

***Corresponding authors**. E-mails: zhaoxinbio@mail.xjtu.edu.cn; baoling@mail.xjtu.edu.cn

[†]Equally contributed to the work.

Results and discussion

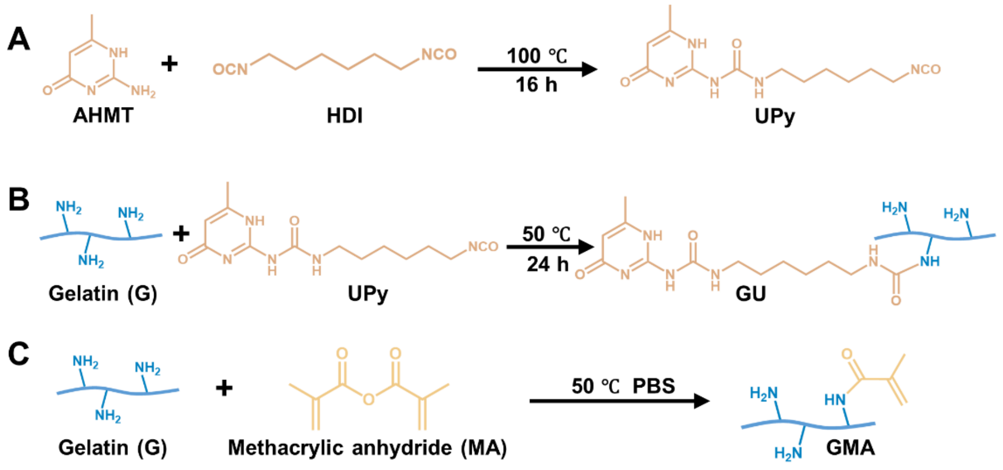

Figure S1. (A, B and C) Synthesis of UPy (A), GU (B) and GMA(C).

Table S1. Final concentrations of components in UNMx/BAy hydrogels

Sample name	GU （mg/mL）	GMA （mg/mL）	NIPAM （mg/mL）	BTO@Au （mg/mL）
UNM0	50	0	100	0
UNM5	50	5	100	0
UNM10	50	10	100	0
UNM20	50	20	100	0
UNM10/BA0.5	50	10	100	0.5
UNM10/BA1	50	10	100	1
UNM10/BA2	50	10	100	2

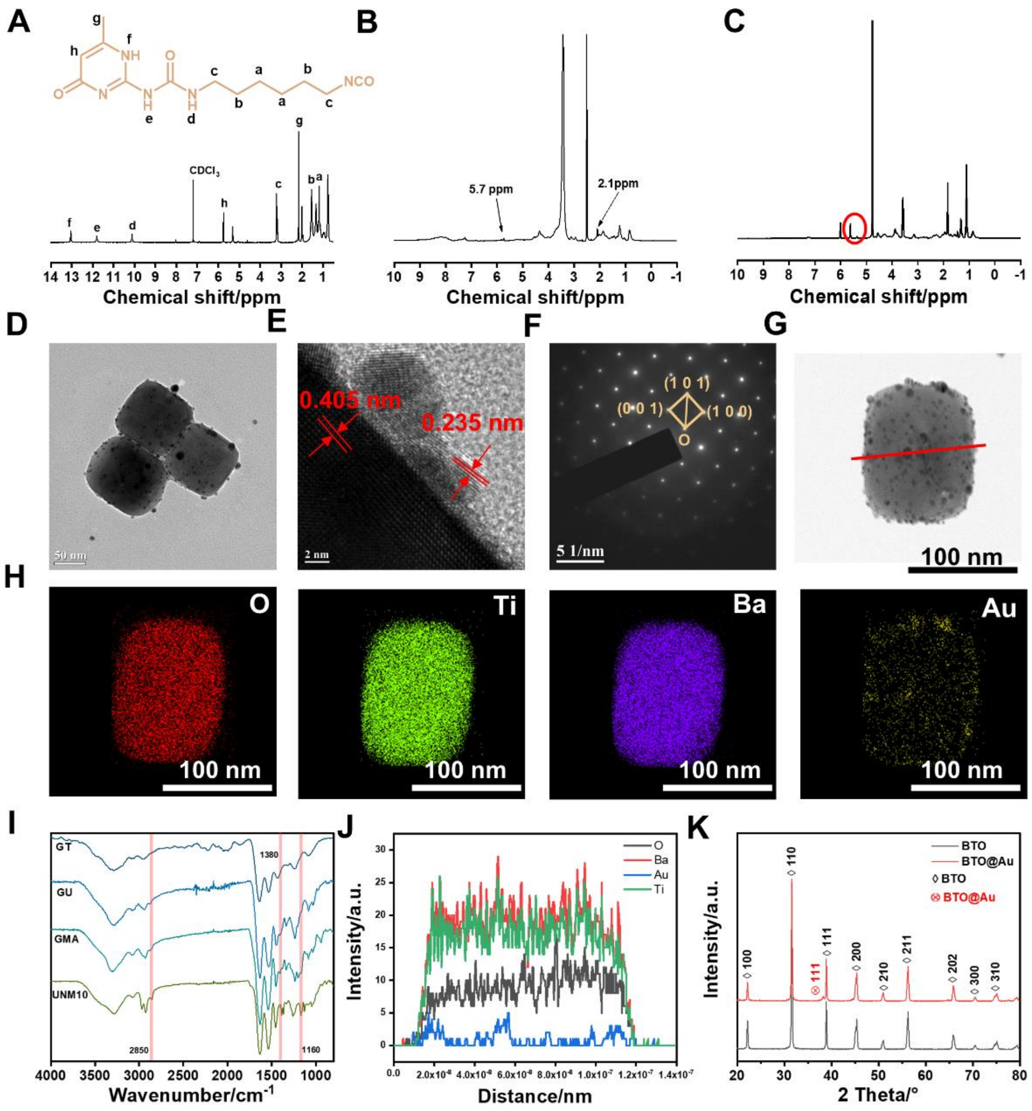

Figure S2. Characterization of UPy, GU, GMA, UNM hydrogel, and BTO@Au. (A) ^{1}H NMR spectrum of UPy. (B) ^{1}H NMR spectrum of GU. (C) ^{1}H NMR spectrum of GMA. (D) TEM image showing the microstructure of BTO@Au. (E) HRTEM image of BTO@Au. (F) Selected area electron diffraction pattern of BTO@Au. (G) EDX line scan of BTO@Au. (H) EDX mapping results of BTO@Au. (I) FT-IR spectra of GT, GU, GMA, and UNM10. (J) EDX line scan results of BTO@Au. (K) XRD patterns of BTO and BTO@Au.

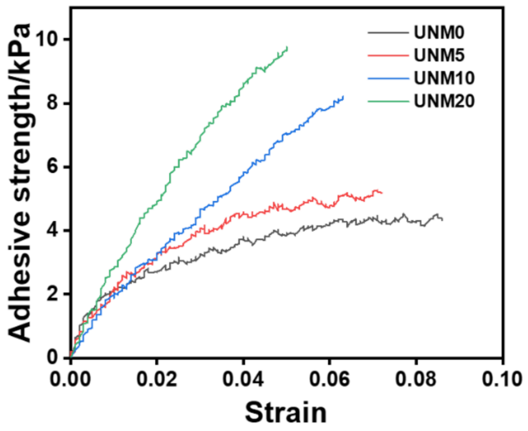

Figure S3. Representative adhesive strength-strain curves of hydrogels.

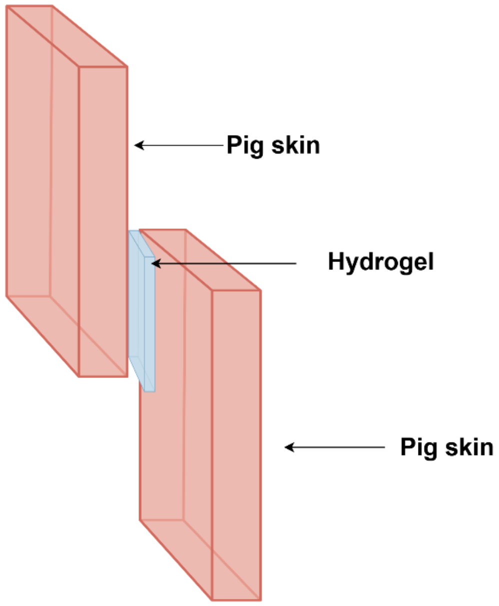

Figure S4. Schematic diagram of hydrogel adhesion experiment.

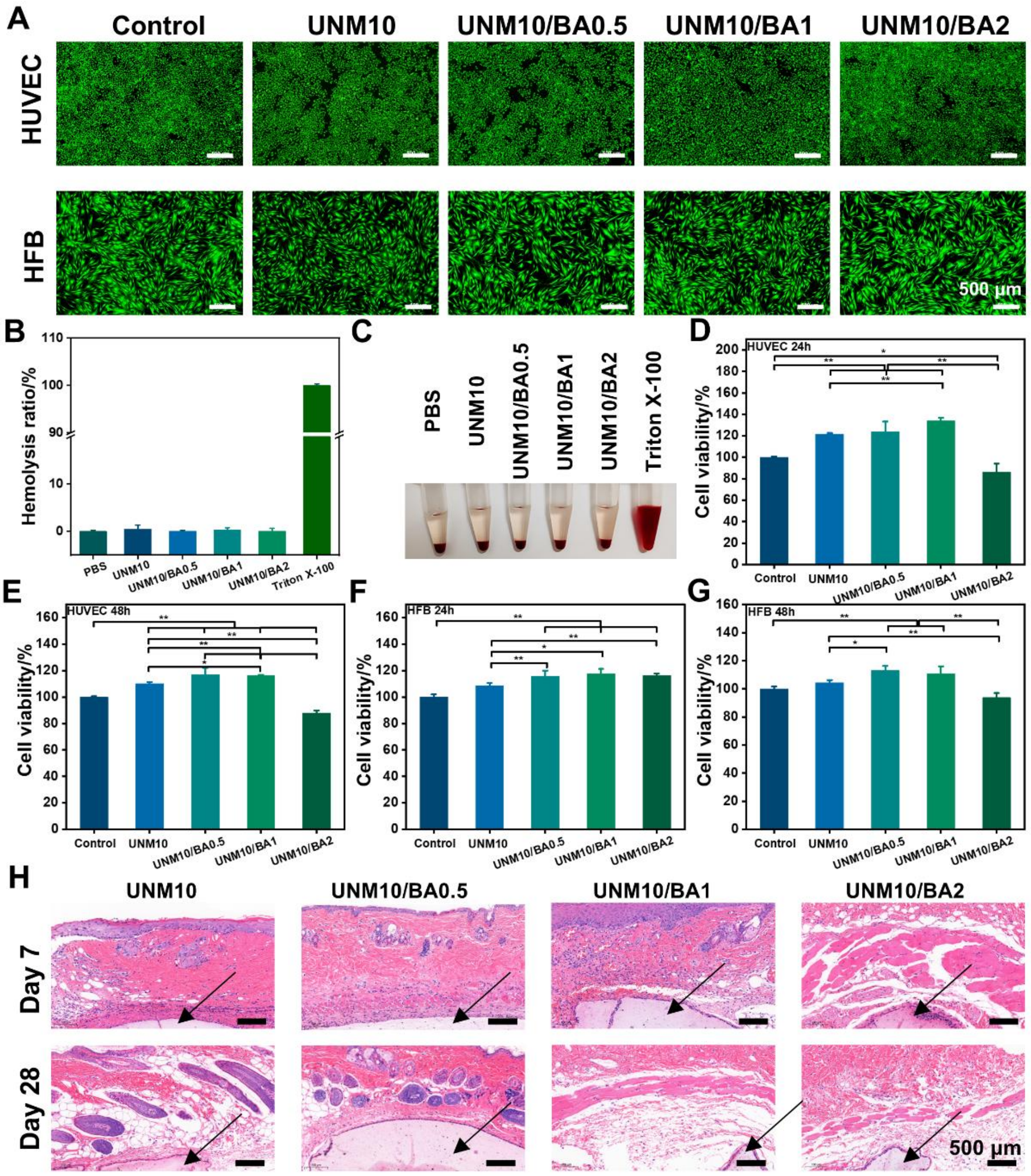

Figure S5. Biocompatibility of the hydrogel. (A) Representative images of LIVE/DEAD® staining of HUVEC and HFB co-cultured with the hydrogel for 24 hours. (B and C) Hemolysis ratio of the hydrogel and representative images of the supernatant after centrifugation. (D and E) Cell viability of HUVEC treated with the hydrogel for 24 hours (D) and 48 hours (E). (F and G) Cell viability of HFB treated with the hydrogel for 24 hours (F) and 48 hours (G). (H) H&E staining images of

adjacent skin tissues after subcutaneous implantation of the hydrogel in rats for 7 and 28 days. *$p < 0.05$, **$p < 0.01$.

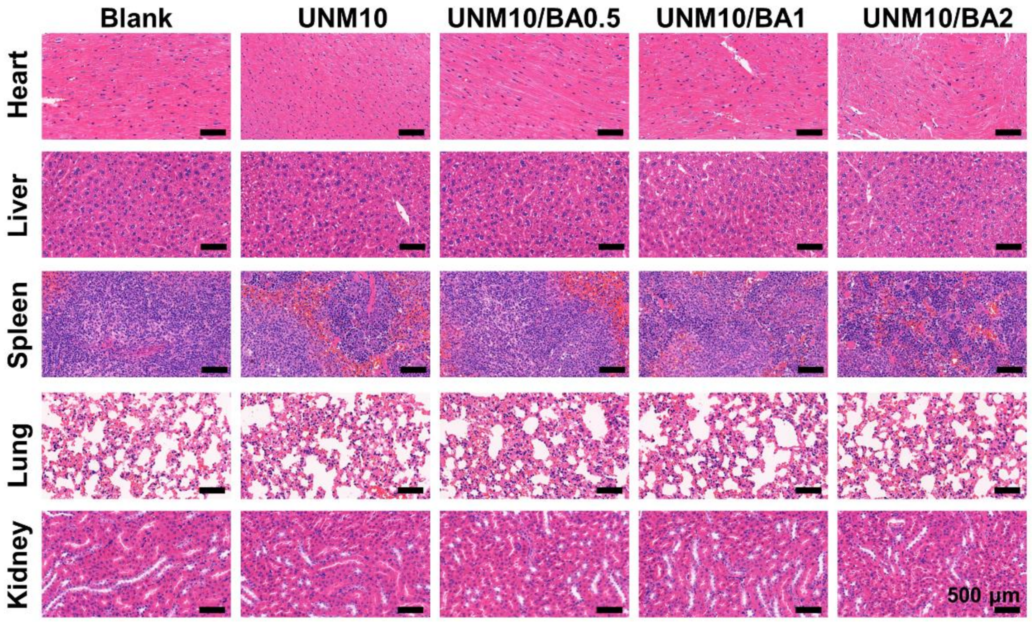

Figure S6. H&E staining images of the biocompatibility of hydrogels *in vivo* with major organs (28 days after treatment).

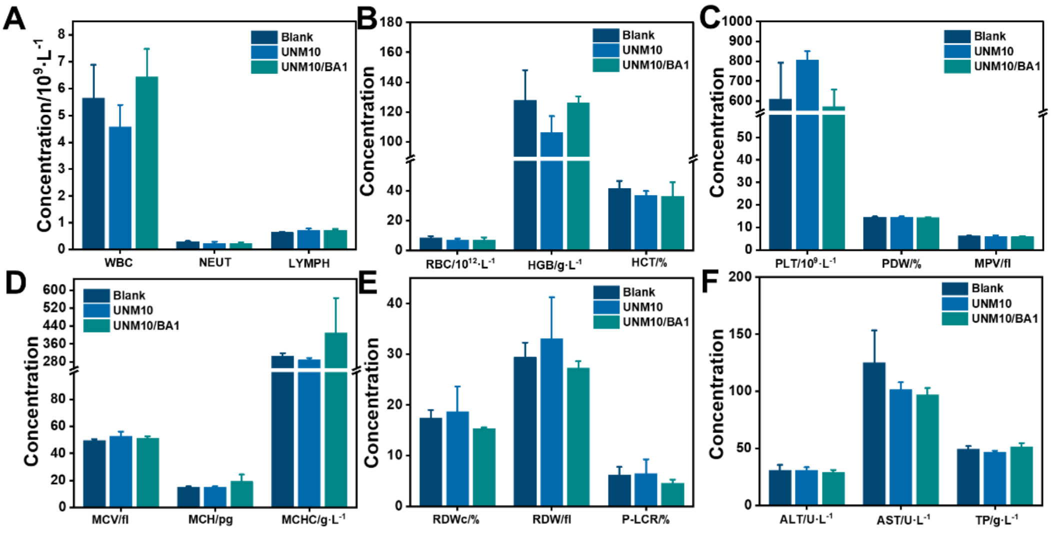

Figure S7. *In vivo* biocompatibility assessment of hydrogels. (A-E) Blood parameters in normal mice with different treatments for 28 days. (F) Serum levels of liver and

kidney function indicators including alanine transaminase (ALT), aspartate transaminase (AST), total protein (TP).

The cytotoxicity of the hydrogel was assessed using Live/Dead® cell staining by co-culturing HUVECs and HFBs with the hydrogel. As shown in Fig. S5A, the majority of HUVEC and HFB cells stained green (live cells) with only a few red (dead cells), indicating good biocompatibility of all four hydrogel dressings.

The *in vitro* biocompatibility was further quantified using hemolysis activity tests. As shown in Fig. S5B, the hemolysis ratio of all groups was below 5%, indicating good blood compatibility. Representative images in Fig. S5C further demonstrate this. Direct contact cytotoxicity tests were performed to address concerns about higher concentrations of leached chemicals near the hydrogel. Hydrogel disks (5 mm diameter, 0.5 mm thickness) were cut and co-cultured with cells, mimicking the contact environment between the hydrogel and wound surface tissue cells. As shown in Fig. S5D, HUVEC cell viability treated with UNM10, UNM10/BA0.5, and UNM10/BA1 was approximately 20% higher than the control on the first day, while UNM10/BA2 showed about a 20% decrease. After two days of incubation, all groups except UNM10/BA2 showed continued proliferation of HUVEC cells, with UNM10/BA0.5 and UNM10/BA1 exhibiting the highest cell viability, up to 118% (Fig. S5E). As shown in Fig. S5F, when co-cultured with HFB cells, the cell viability of all four hydrogel-treated groups increased by approximately 15% on the first day, but the viability of UNM10/BA2 decreased to 95% on the second day, while the other groups remained above 100% (Fig. S5G). These results indicate good

biocompatibility for all four hydrogel dressings (cell viability greater than 80%), but a high concentration of nanoparticles in the hydrogel (UNM10/BA2) is detrimental to cell proliferation.

Subcutaneous implantation experiments in rats were conducted to further characterize the host inflammatory response *in vivo*, evaluated specifically by H&E staining. As shown in Fig. S5H, on the seventh day of implantation, all groups exhibited acute inflammatory responses due to the foreign implants, with noticeable fibrotic inflammation zones around the materials. By the 28th day of implantation, the fibrotic inflammation zones around the hydrogel dressings had significantly narrowed, and the number of inflammatory cells had noticeably decreased. Further analysis of major organs from rats implanted with hydrogel dressings for 28 days, compared to healthy rats as a blank control, showed no significant hemorrhage, congestion, or necrosis in the heart, liver, spleen, lung, or kidney tissues (Fig. S6). Additionally, blood routine test indicators showed no significant differences compared to healthy rats (Fig. S7). These results confirm that the UNMx/BAy hydrogel dressings exhibit excellent *in vitro* and *in vivo* biocompatibility.

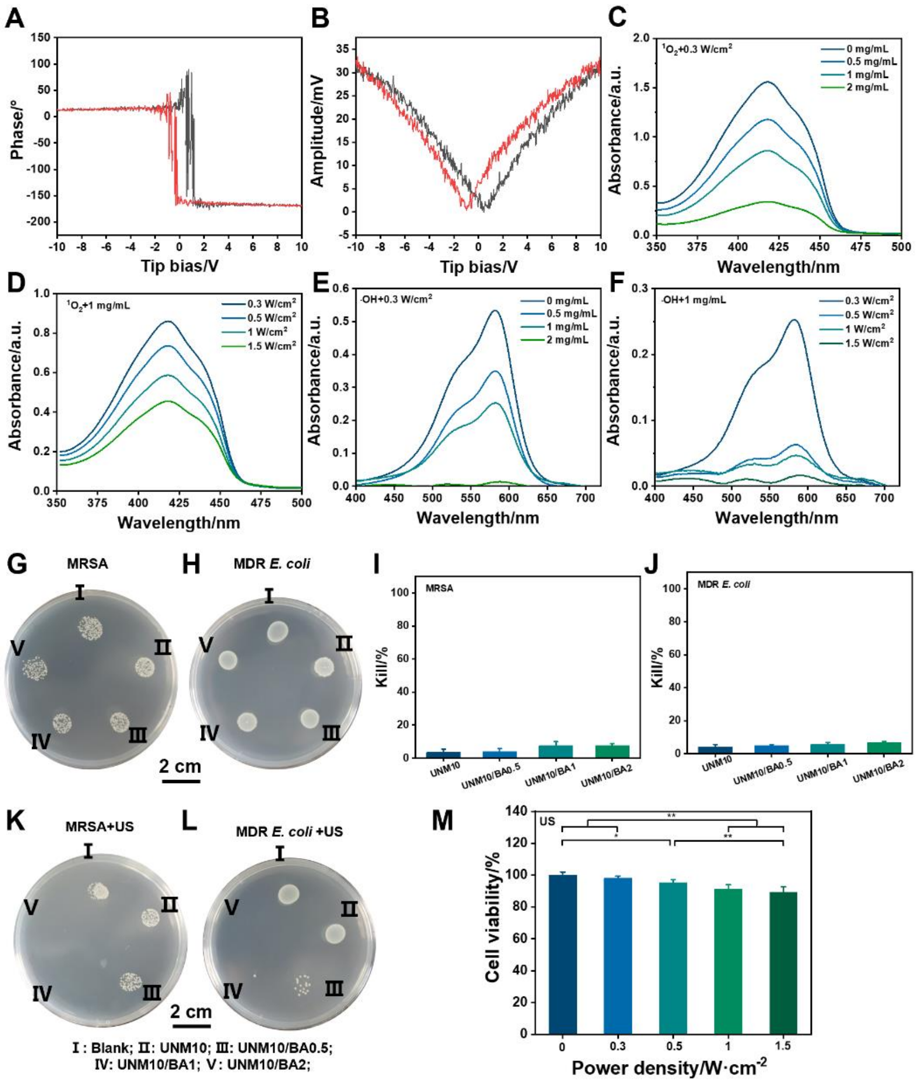

Figure S8. (A) The phase-voltage curve of BTO@Au. (B) Amplitude-voltage curve of BTO@Au. (C) Generation capacity of singlet oxygen under ultrasound stimulation at different concentrations of BTO@Au. (D) Generation capacity of singlet oxygen at 1 mg/mL BTO@Au under different power densities of ultrasound treatment. (E) Generation capacity of hydroxyl radicals under ultrasound stimulation at different concentrations of BTO@Au. (F) Generation capacity of hydroxyl radicals at 1 mg/mL BTO@Au under different power densities of ultrasound treatment. (G, H, I, and J) Representative images and Bactericidal ratios and of hydrogels against MRSA and MDR *E. coli* without ultrasound treatment. (K and L) Representative images of

hydrogels against MRSA (K) and MDR *E. coli* (L) with 1.5 W/cm ^{2} ultrasound treatment. (M) Cell viability under exogenous ultrasound treatment. *$p < 0.05$, **$p < 0.01$.

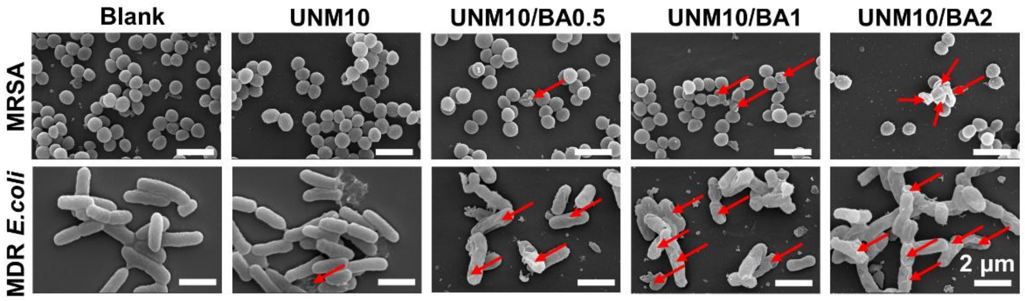

Figure S9. Microscopic morphology of MRSA and MDR *E. coli* after different treatment.

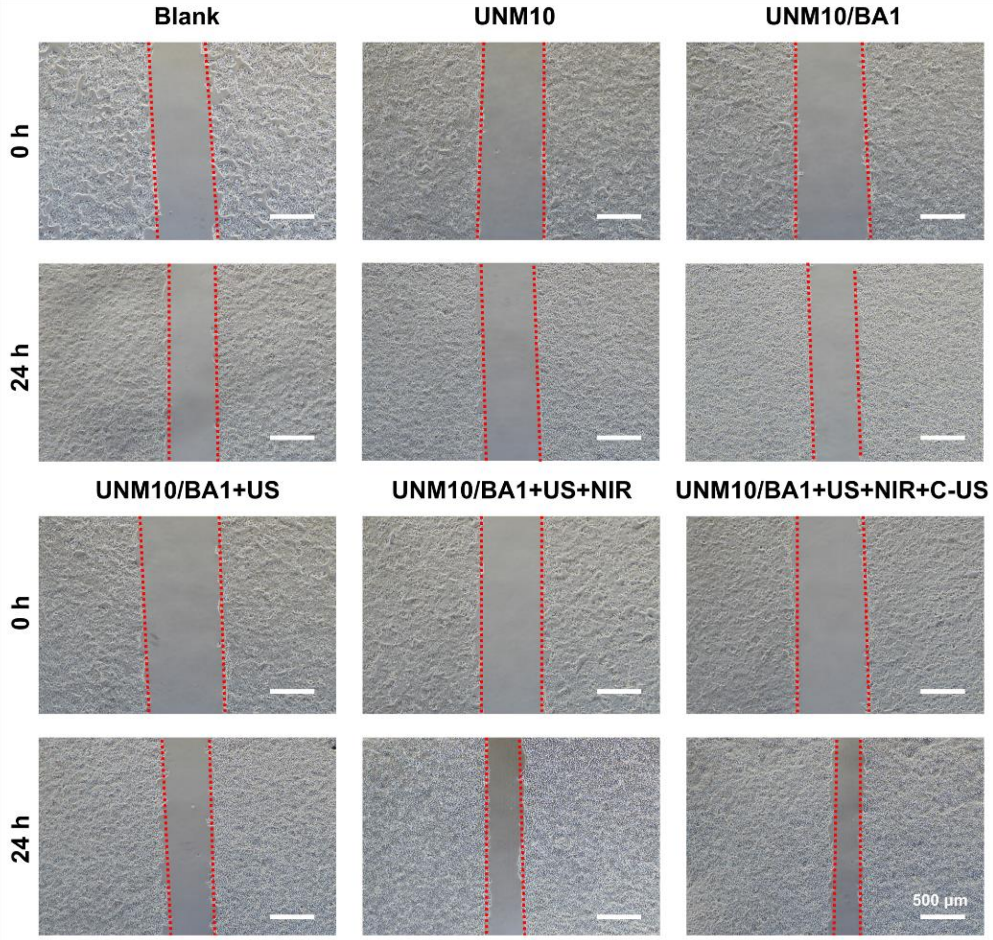

Figure S10. Representative images of *in vitro* scratch assays for HaCaT.

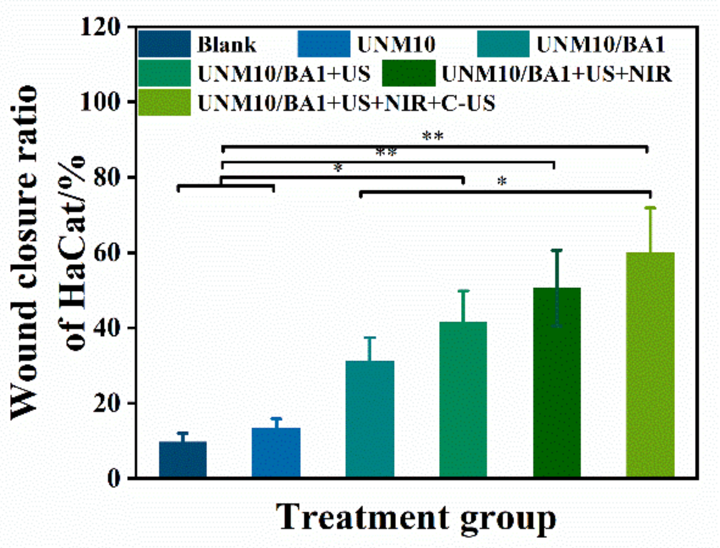

Figure S11. Statistics of wound closure ratio of HaCaT under different treatments. *p < 0.05, **p < 0.01.

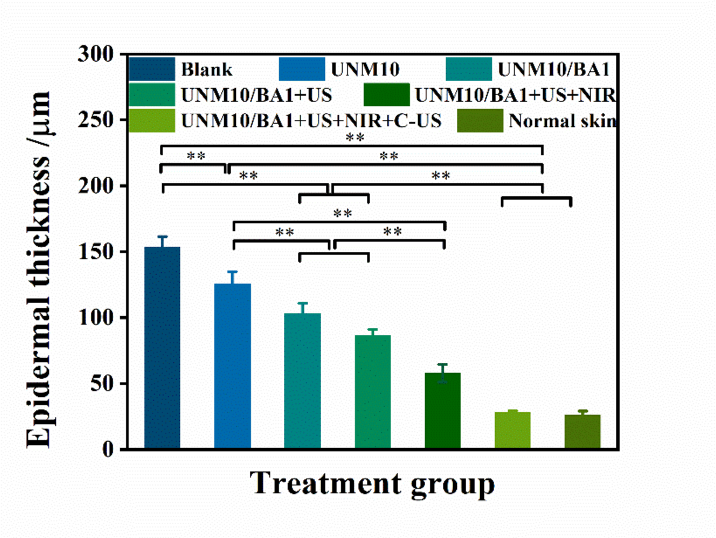

Figure S12. Epidermal thickness from H&E-stained sections on day 15. **p < 0.01.

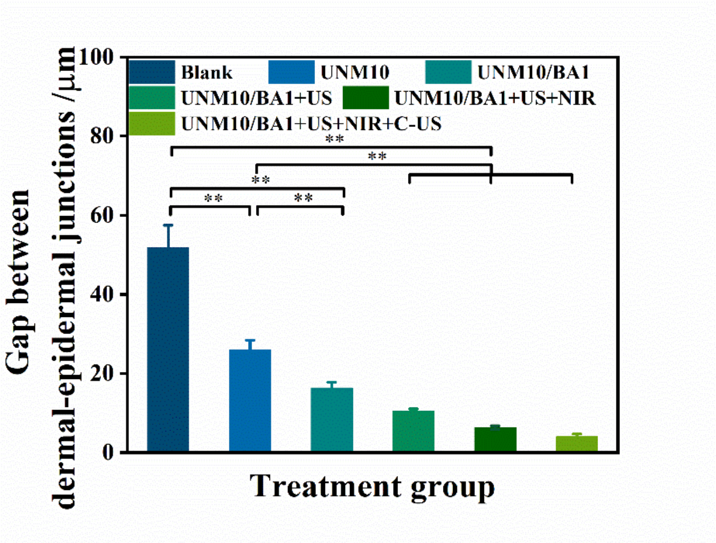

Figure S13. The gap between dermal-epidermal junctions of different treatment group on day 15. $^{**}p < 0.01$.

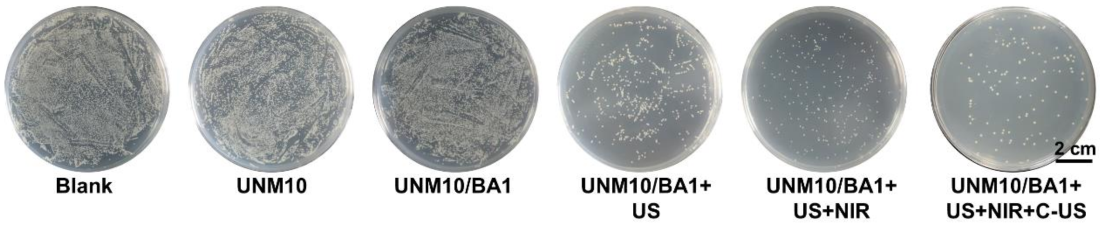

Figure S14. Surviving bacterial colonies in the wound after 5 days of treatment.

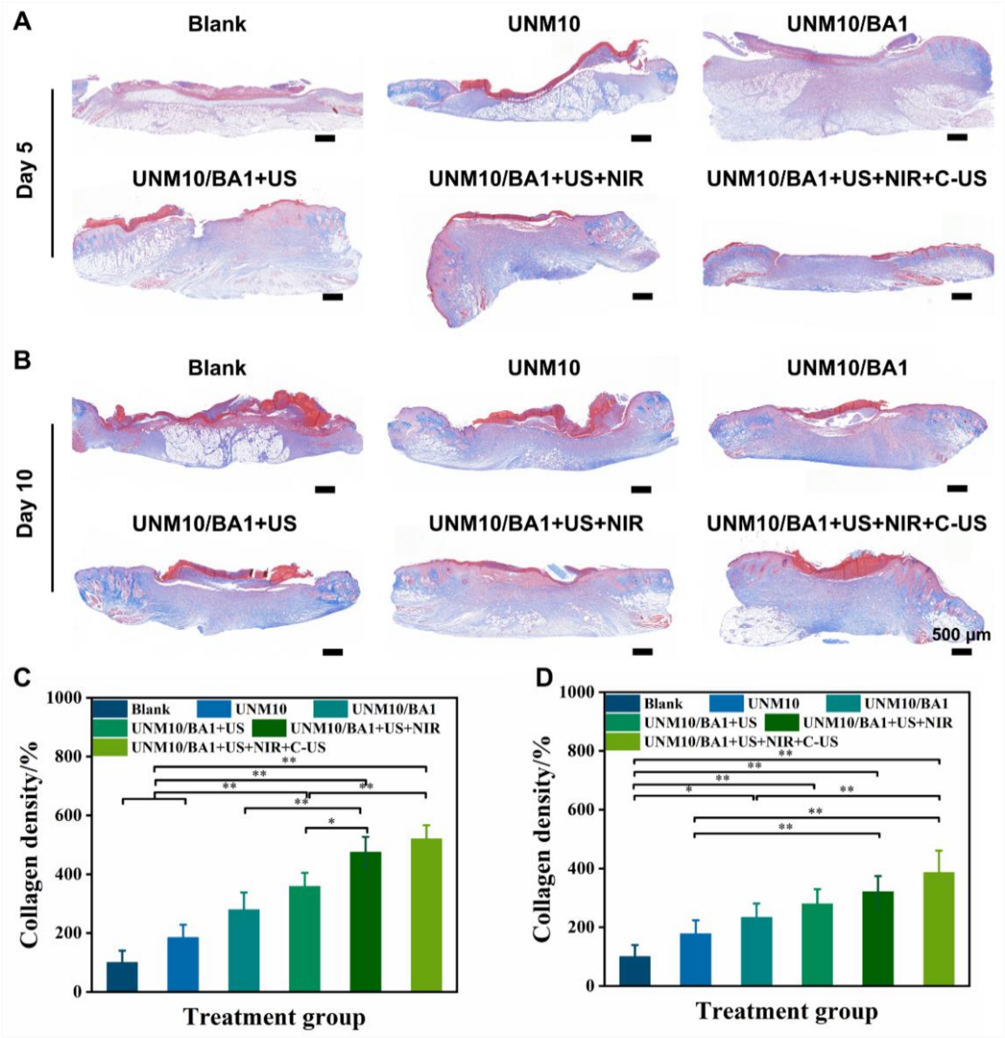

Figure S15. (A-B) Masson's trichrome-stained images on days 5 and 10 for different treatment groups. (C-D) Statistics of collagen deposition at the wound site on days 5 and 10 for different treatment groups. **p* < 0.05, ***p* < 0.01.

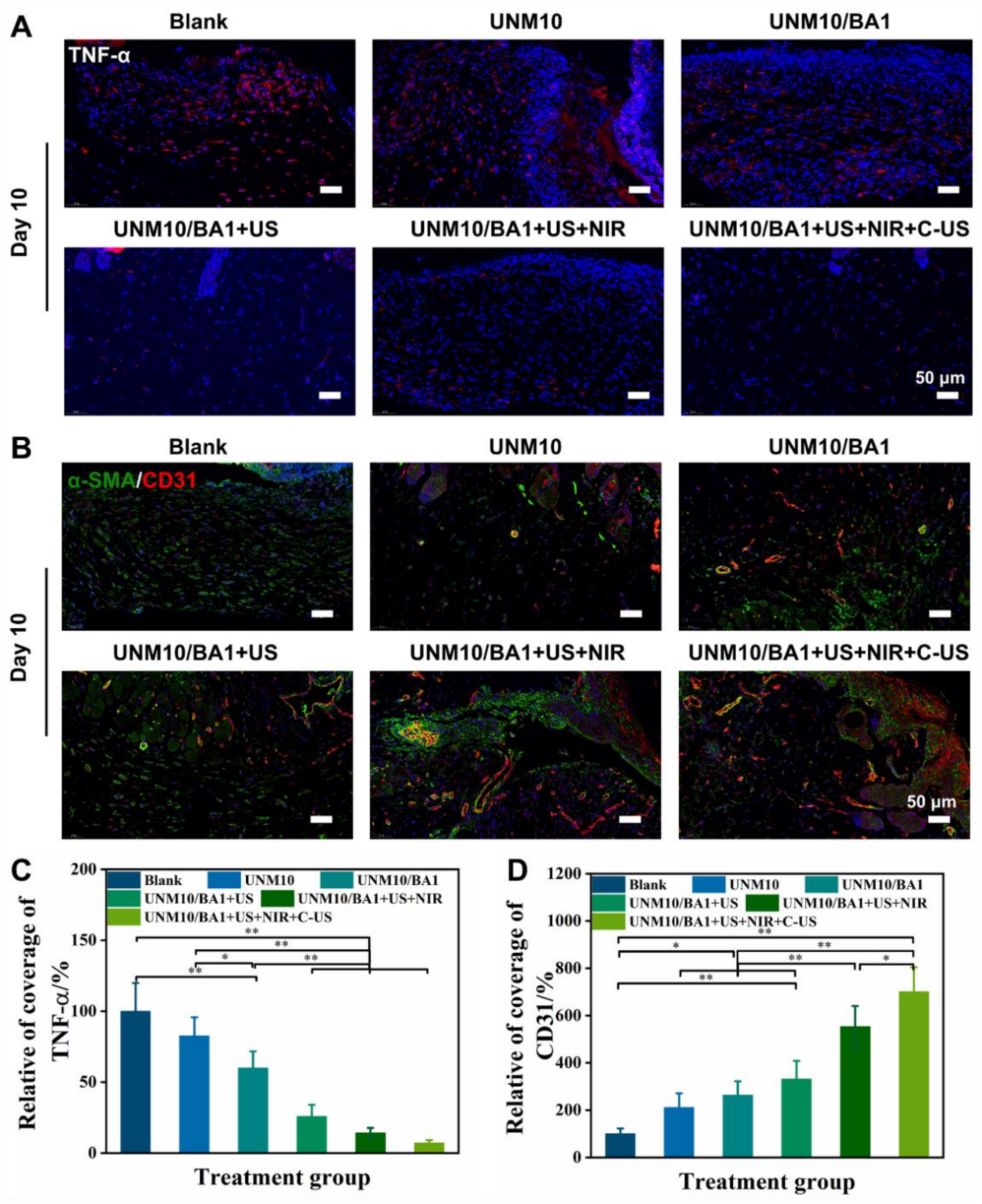

Figure S16. (A-B) Representative images of *in vivo* TNF-α and CD31/α-SMA immunofluorescence staining on day 10. (C) Relative TNF-α expression on day 10. (D) Relative CD31 expression on day 10. $*p < 0.05$, $**p < 0.01$.

Experimental Section/Methods

1. Materials

Gelatin was purchased from Sigma; 2-amino-4-hydroxy-6-methylpyrimidine, barium hydroxide dihydrate, and N-isopropylacrylamide were purchased from

Aladdin; hexamethylene diisocyanate, methacrylic anhydride, tetrabutyl titanate, and ammonia solution (25% v/v) were purchased from J&K Scientific; and chloroauric acid trihydrate was purchased from Alfa Aesar (China). All other reagents were used as received.

2. Synthesis of GU and GMA

GU was synthesized according to a previously reported method[1]. First, ureidopyrimidinone (UPy) was synthesized by reacting 2-amino-4-hydroxy-6-methylpyrimidine (AHMT) with hexamethylene diisocyanate (HDI), as shown in Fig. S1A. The synthesis of GU is illustrated in Fig. S1B, in which UPy is modified by reacting its isocyanate groups with the amino groups on gelatin. Specifically, 2 g of type A porcine skin gelatin was dissolved in 25 mL of anhydrous dimethyl sulfoxide (DMSO) at a concentration of 8 wt %. Simultaneously, 150 mg of UPy was dissolved in 5 mL of anhydrous DMSO, and this solution was then added dropwise to the gelatin-DMSO solution under continuous stirring. After the reaction at 50 ℃ for 24 hours, the resulting mixture was precipitated in diethyl ether. The precipitate was redispersed in deionized water and dialyzed using dialysis bags with a molecular weight cutoff of 10,000 Da for 7 days. The final product, GU, was obtained by lyophilization.

GMA was synthesized according to a previously reported method[2]. It was synthesized as shown in Fig. S1C. Ten grams of type A porcine skin gelatin was dispersed in 0.01 M phosphate-buffered saline (PBS) at a concentration of 10 wt % and dissolved by stirring at 60 ℃ using a magnetic stirrer. After complete dissolution,

8 mL of methacrylic anhydride was added to the gelatin solution, and the mixture was stirred continuously at 50 ℃ for 3 hours. Following the reaction, the polymer solution was diluted fivefold with warm (40 ℃) 0.01 M PBS to terminate the reaction. The mixture was then dialyzed in deionized water at 40 ℃ for 7 days using dialysis bags (molecular weight cutoff 10,000 Da) to remove salts and unreacted methacrylic anhydride. The final product, GMA, was obtained by lyophilizing the dialyzed solution.

3. Synthesis of BTO@Au Nanoparticles

Barium titanate nanocubes were synthesized according to a previously reported method[3], using barium hydroxide dihydrate and tetrabutyl titanate as starting materials. A mixture of 17.018 g (50 mmol) of tetrabutyl titanate and 20 mL of ethanol was prepared, to which 7 mL of 25 wt% ammonia solution was added, forming system A. Separately, 14.204 g (50 mmol) of barium hydroxide dihydrate was dissolved in 25 mL of deionized water to obtain a clear solution, which was then added to system A to form a homogeneous suspension B. Suspension B was then transferred to a 100 mL polytetrafluoroethylene-lined autoclave and subjected to a hydrothermal reaction at 200 ℃ for 48 hours. The product obtained was repeatedly washed with acetic acid and anhydrous ethanol and then dried in an oven at 80 ℃ to yield barium titanate nanocubes.

The synthesis of BTO@Au was based on a modified existing method[4]. To synthesize BTO@Au, first, 1.6 mL of 25% ammonia solution was diluted with deionized water to pH 11, and 20 mM barium titanate nanocubes (4.664 g) were

dispersed in this solution under continuous stirring to form suspension C. Next, 4 mL of a 10 mg/mL chloroauric acid trihydrate solution was prepared and added to suspension C to form suspension D, which was then vigorously stirred at 80 ℃ for 1 hour. After the reaction, the product was repeatedly washed with deionized water and ethanol, and dried. Finally, the product was calcined in a muffle furnace at 300 ℃ for 1 hour and allowed to cool to room temperature to obtain the final BTO@Au nanocomposite.

4. Preparation of UNMx/BAy hydrogels

A 200 mg/mL GMA solution, a 333.33 mg/mL N-isopropylacrylamide (NIPAM) solution, a 250 mg/mL ammonium persulfate (APS) solution, and a 50 μL/mL N,N,N',N'-tetramethylethylenediamine (TEMED) solution were prepared. GU was dissolved in deionized water at a concentration of 100 mg/mL by heating at 60 ℃. BTO@Au was dispersed in deionized water at a concentration of 50 mg/mL, followed by ultrasonication for 30 minutes to obtain a stable suspension. A mixture of 500 μL GU solution, 300 μL NIPAM solution, and a specific amount of GMA solution was prepared in a centrifuge tube. Subsequently, 20 μL APS solution and 20 μL TEMED solution were rapidly added with shaking. After sufficient reaction, the hydrogel UNMx was obtained, where x represents the final concentration of GMA in the hydrogel (in mg/mL). For the preparation of UNMx/BAy hydrogels, an appropriate amount of BTO@Au suspension was added before the addition of APS and TEMED solutions. Here, BA represents BTO@Au, and y denotes the final concentration of BTO@Au in the hydrogel (in mg/mL). Detailed parameters for the preparation of

hydrogel dressings are listed in Table S1.

5. Characterizations

Proton nuclear magnetic resonance (^{1}H NMR, 400 MHz) spectra of UPy, GU, and GMA were recorded using a Bruker Ascend instrument. D_2O served as the solvent and internal standard for GMA, while DMSO-d6 was used for UPy and GU. Fourier transform infrared (FT-IR) spectra were acquired using a Nicolet 6700 FT-IR spectrometer (Thermo Scientific Instrument). FT-IR spectra of freeze-dried gelatin, GU, GMA, and UNM10 hydrogels were recorded in the range of 4000-650 cm^{-1}. The microstructure, high-resolution images, and selected area electron diffraction patterns of BTO@Au were acquired via a transmission electron microscope (TEM; JEM-2100F, JEOL, Japan) operating at 200 kV. Elemental distribution was characterized through energy-dispersive X-ray spectroscopy (EDX) equipped with the TEM. The phase composition of BTO and BTO@Au samples was determined by X-ray diffraction (XRD; X'Pert PRO, Netherlands).

6. Rheological Testing of Hydrogels

The rheological properties of the prepared hydrogels (20 mm in diameter) were tested using a TA rheometer (DHR-2) in frequency sweep mode. Measurements were conducted at 25 ℃, with 1% strain and a frequency range of 0.1-100 rad/s. Additionally, temperature-dependent rheological properties of the prepared hydrogels (20 mm in diameter) were tested in temperature sweep mode, with data recorded under 1% strain and 10 rad/s frequency over a temperature range of 25-50 ℃.

7. Thermo-induced shrinkage performance of hydrogels

The thermo-induced shrinkage performance of the hydrogels was tested in a water bath at 37 ℃. Hydrogel samples were prepared as disc-shaped specimens with a bottom diameter of approximately 8 mm and a height of 0.5 mm. The initial diameter of the samples was recorded before the test. The samples were then placed in deionized water at 37 ℃ and heated in the water bath for a predetermined duration. After heating, the samples were removed, and their shrunken diameters were measured using a vernier caliper. Subsequently, the water bath temperature was raised to 45 ℃, and the hydrogel samples were allowed to shrink for a certain period. The shrunken diameter of the samples was recorded after incubating in the 37 ℃ water bath for 3 minutes. Each experiment was repeated three times with different samples. The thermo-induced shrinkage strain was calculated using the following formula: Shrinkage strain $(\%) = 100\% - (\frac{d_1}{d_2})^2 \times 100\%$, where d_1 is the diameter after shrinkage and d_2 is the initial diameter of the sample.

8. Uniaxial Compression Testing of Hydrogels

The uniaxial compressive stress-strain curves of the hydrogels were measured using an Instron material testing system (MTS Criterion 43, MTS Criterion) equipped with a 50 N load cell at room temperature. The compression speed was set at 2 mm/min. All samples were prepared as cylinders with a height of 7000 μm and a base diameter of 5 mm.

9. Tensile Performance Testing of Hydrogels

The tensile stress-strain curves of the hydrogels were obtained using an Instron material testing system (MTS Criterion 43, MTS Criterion) equipped with a 50 N load

cell at room temperature. The tensile speed was set at 2 mm/min. All samples were prepared as rectangular specimens with a length of 30 mm and a width of 6 mm.

10. Adhesion Strength Testing of Hydrogels to Tissue

The adhesion strength of the hydrogels to tissue was assessed via a universal tensile testing machine. Freshly excised pig skin was cut into rectangular strips (10 mm × 30 mm). Fifty microliters of hydrogel precursor were evenly applied onto the surface of the freshly excised skin tissue, and another piece of skin was overlaid on top of the hydrogel, maintaining a contact area of 10 mm × 10 mm between the two skin tissues. The pig skin and hydrogel precursor were crosslinked at 37 ℃ and high humidity for 3 hours. The adhesion strength of the hydrogel to skin tissue was tested using a lap shear test on an Instron material testing system (MTS Criterion 43, MTS Criterion) equipped with a 50 N load cell. The tensile speed was set at 5 mm/min. Each experiment was repeated five times with different samples.

11. Microstructural Characterization of Hydrogels

The microstructure of the freeze-dried hydrogels was examined through a field emission scanning electron microscope (FEI Quanta FEG 250). The pore size of the hydrogels was calculated using ImageJ software. The hydrogels were prepared as cylindrical samples with a diameter of 5 mm and fully freeze-dried to ensure no moisture remained inside, then sliced into 1 mm thick sections. Before observation, the hydrogel surface was gold-coated.

12. Conductivity Testing of Hydrogels

The electrical conductivity of the hydrogels was assessed through an Agilent

digital multimeter. Rectangular specimens were prepared. Each experiment was repeated three times, and the conductivity was determined using the formula: $\text{Conductivity}\,(\%) = \frac{L}{R \times S} \times 100\%$, where R is the measured resistance, L is the length of the sample, and S is the bottom area of the sample.

13. Piezoelectric and Sonodynamic Performance Testing of Hydrogels

The phase-voltage and amplitude-voltage curves of BTO@Au were measured using a piezoresponse force microscope (PFM; ICON, Bruker, USA). The singlet oxygen ($^1O_2$) generation capability of BTO@Au was characterized using 1,3-diphenylisobenzofuran (DPBF) as an indicator, where $^1O_2$ caused a decrease in absorbance at 418 nm. All experiments were conducted in the dark. DPBF solutions at a concentration of 100 μM were prepared in dimethyl sulfoxide (DMSO), and BTO@Au was dispersed in these solutions at concentrations of 0.5 mg/mL, 1 mg/mL, and 2 mg/mL, then stirred for 60 minutes to reach adsorption-desorption equilibrium. The dispersion was transferred to 24-well plates at a volume of 2 mL per well and subjected to ultrasound at different power densities (0.3 W/cm^2; 0.5 W/cm^2; 1 W/cm^2; 1.5 W/cm^2) for 1 minute. After treatment, the supernatant was collected, and the residual DPBF absorbance spectra were measured using a UV-Vis spectrophotometer over the range of 200-800 nm. The hydroxyl radical (·OH) generation capability of BTO@Au was characterized using methyl violet (MV) as an indicator, where ·OH caused a decrease in absorbance at 583 nm. All experiments were conducted in the dark. MV solutions at a concentration of 10 μM were prepared in deionized water, and BTO@Au was dispersed in these solutions at concentrations of 0.5 mg/mL, 1 mg/mL,

and 2 mg/mL, then stirred for 60 minutes to reach adsorption-desorption equilibrium. The dispersion was transferred to 24-well plates at a volume of 2 mL per well and subjected to ultrasound at different power densities (0.3 W/cm^2, 0.5 W/cm^2, 1 W/cm^2, 1.5 W/cm^2) for 1 minute. After treatment, the supernatant was collected, and the residual MV absorbance spectra were measured using a UV-Vis spectrophotometer over the range of 200-800 nm. Each experiment was repeated three times with different samples.

14. Hemolysis Activity Testing of Hydrogels

For hemolysis activity testing, fresh mouse blood anticoagulated with sodium heparin was centrifuged at 1000 rpm to collect red blood cells (RBCs), which were then washed three times with PBS and diluted 20 times with PBS. The hydrogel samples (500 μL) were prepared in 24-well plates, and 500 μL of RBC dispersion was added to each well. After incubation at 37 ℃ for 1 hour, the RBC dispersion was collected and centrifuged at 1000 rpm for 10 minutes. The supernatant (100 μL) was transferred to a 96-well plate, and the absorbance at 540 nm was recorded using a microplate reader (Molecular Devices). PBS was used as the negative control, and Triton X-100 was used as the positive control. The hemolysis ratio (%) was calculated using the formula: Hemolysis ratio $(\%) = \frac{A_h - A_b}{A_t - A_b} \times 100\%$, where A_h is the absorbance of the supernatant treated with hydrogel samples, A_b is the absorbance of the PBS-treated supernatant, and A_t is the absorbance of the Triton X-100-treated supernatant.

15. *In vitro* cytocompatibility testing of hydrogels

The complete growth medium was Dulbecco's Modified Eagle Medium (DMEM) (Gibco), supplemented with 10% fetal bovine serum (Gibco), 1.0×10^5 U/L penicillin (Hyclone), and 100 mg/L streptomycin (Hyclone). Sterilized pre-soaked hydrogels were cut into discs with a diameter of 5 mm and a height of approximately 500 μm. Human skin fibroblasts (HFB) and human umbilical vein endothelial cells (HUVEC) were seeded into 96-well plates at a density of 10,000 cells per well. After 24 hours of incubation, the hydrogel discs were added to each well. After 24 and 48 hours of incubation, the eluate or hydrogel discs and the culture medium were removed, and cell viability was detected using a CCK-8 assay kit according to the manufacturer's instructions. Additionally, a LIVE/DEAD® assay kit was used to evaluate the viability of cells co-cultured with the hydrogel discs. After 24 hours of co-culture, the hydrogel discs and culture medium were removed, and the LIVE/DEAD® assay reagent was added according to the instructions. After incubation, images were taken using a fluorescence microscope (Nikon, DS-Ri2, Japan).

16. Cell Proliferation Assay and *In Vitro* Wound Healing Assay

For the cell proliferation assay, the complete growth medium was Dulbecco's Modified Eagle Medium (DMEM) supplemented with 10% fetal bovine serum, 1.0×10^5 U/L penicillin, and 100 mg/L streptomycin. Sterilized pre-soaked hydrogels were cut into discs with a diameter of 5 mm and a height of approximately 500 μm. Human fibroblast cells (HFB) were seeded into 96-well plates at a density of 10,000 cells per well. After 24 hours of incubation, the hydrogel discs were added to each well and subjected to ultrasound treatment at different power densities (0.3 W/cm^2,

0.5 W/cm^2, 1 W/cm^2, and 1.5 W/cm^2) for 1 minute, with a non-ultrasound group serving as the control. After 24 hours of co-culture, the hydrogel discs and culture medium were removed, and cell viability was detected using a CCK-8 assay kit.

The effect of ultrasound treatment on cell proliferation and migration was additionally evaluated through a scratch assay on HFB cells. Cells were seeded into the lower chamber at a density of 2×10^5 cells per well, and hydrogel discs were placed in the upper chamber. Multiple scratches were made on the culture plate surface using a sterile pipette tip. The healing process was observed under a microscope (IX53, Olympus) after a certain period.

The scratch assay for HaCaT cells was performed in 6-well plates (LABSELECT). Briefly, cells were passaged at a density of 2×10^5 cells per well. Once the cells reached confluence, multiple scratches were made on the culture plate surface using a sterile pipette tip. Images of the scratches were taken at 0 and 24 hours using a fluorescence microscope, and the wound closure rate was calculated as the ratio of the closed area to the initial wound area.

17. *In Vivo* Implantation Test of Hydrogels

All animal experiments were conducted under the guidelines of the Animal Research Committee of Xi'an Jiaotong University. SD rats (female, 250 g) were anesthetized by isoflurane anesthesia. Sterilized hydrogels (100 μL) were implanted subcutaneously in the rats. After 7 and 28 days, the rats were euthanized, and tissue samples were collected for histological analysis. Blood routine tests were performed on the animals before tissue removal. The inflammatory response was measured using

hematoxylin and eosin (H&E) staining. The inflammatory state of the grouped tissues and the health status of the organs were observed under a microscope.

18. *In Vitro* Antibacterial Activity Test of Hydrogel Dressings

The release of antibacterial agents was assessed through plate counting. Ten microliters of multidrug-resistant *Escherichia coli* (MDR *E. coli*) and methicillin-resistant *Staphylococcus aureus* (MRSA) suspensions (10^8 CFU/mL) were suspended in each well containing 490 μL of sterile PBS in a 48-well plate. Subsequently, the hydrogel discs were placed in the bacterial suspension, and the plate was pre-cooled with ice for 5 minutes before further processing. Each well was treated with different power densities of ultrasound according to the set protocol, with a control group without hydrogel dressings. After incubation at 37 ℃ for 2 hours, the hydrogel discs were removed, and any surviving bacteria were resuspended. Finally, 10 μL of the resuspension was plated on agar plates and incubated at 37 ℃ for 24 hours to count the colonies. The antibacterial ratio was determined according to the formula: Antibacterial ratio $(\%) = \frac{C_c - C_h}{C_c} \times 100\%$, where C_c is the number of bacterial colonies in the control group, and C_h is the number of bacterial colonies in the hydrogel group.

19. Hydrogel Dressing to Promote Bacterial Infection-Induced Wound Healing Test

All animal experiments were approved by the Animal Research Committee of Xi'an Jiaotong University. Kunming mice (35-40 g, female) were used for the experiment. A full-thickness skin defect wound infected with MRSA was established

on the necks of the mice. The mice were anesthetized by isoflurane anesthesia, and a full-thickness wound with a diameter of 8 mm was created in the central neck area. Ten microliters of 10^8 CFU/mL MRSA (PBS suspension) were added to the wound. After one day, the modeling of the infected wound was completed. The wounds were treated with different methods, and Tegaderm Film was used as a control. To monitor the wound area, photographs of the wound area were taken on days 5, 10, and 15. The wound healing ratio was calculated using the following formula: Wound Healing Ratio (%) = (S_a - S_n)/ S_a×100%, where S_a is the initial wound area, and S_n is the wound area on day n (n = 5, 10, 15). Additionally, skin samples were collected, fixed in 4% paraformaldehyde for 1 hour, embedded in paraffin, and sectioned into 4 μm slices. The obtained sections were stained with H&E and Masson staining (day 5, 10, 15). Immunofluorescence staining of the regenerated skin at the wound site was also performed on day 5 and day 10. The fixed and frozen sections were stained with a dihydroethidium (DHE) probe, TNF-α (Affinity Biosciences), CD31 (Servicebio), α-SMA (Servicebio) and VEGF (Affinity Biosciences). Nuclei were stained with a DAPI-containing sealing solution. All sections were analyzed and photographed under a microscope (IX53, Olympus). Furthermore, a laser speckle blood flow imaging system (RFLSL ZW, RWD Life Science Co., Ltd) was used to detect the blood perfusion status of the wound area on day 5 of treatment.

20. Transcriptome Analysis

On day 10 of treatment, wound tissues from the Blank and UNM10/BA1+US+NIR+C-US groups were collected. Total RNA was extracted using

Trizol reagent (Invitrogen, USA), and RNA quality and concentration were confirmed using a NanoDrop 2000c spectrophotometer (Thermo, USA). Libraries were prepared from pure RNA samples and sequenced using the Illumina HiSeq X10 (Illumina, USA). For transcriptome analysis, bioinformatics data were analyzed on the free online platform of Majorbio Cloud Platform (Majorbio Bio-pharm Biotechnology, China). Sequencing quality was assessed using Fastx-Toolkit v.0.0.14. All reads were mapped to the reference genome of Mus musculus v.GRCm38.p6. Differential expression analysis was performed using DESeq2 v.1.24.0 (cutoff: fold change ⩾1.5 and *p*-value <0.05). Volcano plots were generated using the "EnhancedVolcano" R package. Heatmaps were generated using the "heatmap" function of R. KEGG functional enrichment analysis was performed using KOBAS v.2.1.1.

21. Western Blotting

For Western blotting, protein samples were lysed in RIPA Lysis Buffer with a 1:100 dilution of protease inhibitor cocktail (P1005, Beyotime) and centrifuged at 12,000 rpm for 10 minutes. Supernatants were stored at -80 °C. Protein concentrations were measured using a BCA Protein Kit (Beyotime, USA). Proteins were separated by 10% SDS-PAGE and transferred to PVDF membranes (Millipore, USA). Membranes were blocked with 5% BSA (Millipore, USA) for 1 hour, then incubated overnight at 4 °C with primary antibodies in 5 ml blocking buffer, followed by 1 hour with secondary antibodies at room temperature. Membranes were normalized with anti-β-actin, and bands were detected using an Ultra High Sensitivity ECL Kit (MCE, 5 ml). Imaging was performed on a ChemiScope 3000mini System (Clinx) with automated exposure

settings. Data represent the average of three biological replicates unless otherwise noted.

22. Statistical analysis

Statistical significance was determined using one-way or two-way ANOVA with GraphPad Prism 7.0 software. Data are presented as the mean ± SD of at least three independent measurements. p-value < 0.05 was considered statistically significant.

Reference

1. Zhao X, Liang YP, Huang Y *et al.* Physical double-network hydrogel adhesives with rapid shape adaptability, fast self-healing, antioxidant and nir/ph stimulus-responsiveness for multidrug-resistant bacterial infection and removable wound dressing. *Adv Funct Mater*. 2020; **30**(17): 1910748. doi: 10.1002/adfm.201910748
2. Chen JY, He JH, Yang YT *et al.* Antibacterial adhesive self-healing hydrogels to promote diabetic wound healing. *Acta Biomater*. 2022; **146**: 119-130. doi: 10.1016/j.actbio.2022.04.041
3. Zhu P, Chen Y, Shi JL. Piezocatalytic tumor therapy by ultrasound-triggered and BaTiO3-Mediated piezoelectricity. *Adv Mater*. 2020; **32**(29): 2001976. doi: 10.1002/adma.202001976
4. Wu M, Zhang Z, Liu Z *et al.* Piezoelectric nanocomposites for sonodynamic bacterial elimination and wound healing. *Nano Today*. 2021; **37**: 101104. doi: 10.1016/j.nantod.2021.101104
